# HCN channels in the lateral habenula regulate pain and comorbid depressive‐like behaviors in mice

**DOI:** 10.1111/cns.14831

**Published:** 2024-07-03

**Authors:** Xue‐zhong Cao, Meng‐ye Zhu, Gang Xu, Fan Li, Yi Yan, Jin‐jin Zhang, Jianbing Wang, Fei Zeng, Yang Bao, Xue‐xue Zhang, Tao Liu, Da‐ying Zhang

**Affiliations:** ^1^ Department of Pain Medicine, the First Affiliated Hospital, Jiangxi Medical College Nanchang University Nanchang Jiangxi China; ^2^ Key Laboratory of Neuropathic Pain, the First Affiliated Hospital, Jiangxi Medical College, Nanchang University Healthcare Commission of Jiangxi Province Nanchang Jiangxi China; ^3^ Jiangxi Key Laboratory of Pain Medicine, the First Affiliated Hospital, Jiangxi Medical College Nanchang University Nanchang Jiangxi China; ^4^ Department of Anesthesiology Jiangxi Cancer Hospital Nanchang Jiangxi China; ^5^ Department of Pediatrics the First Affiliated Hospital, Jiangxi Medical College, Nanchang University Nanchang Jiangxi China

**Keywords:** comorbid anxiodepressive‐like symptoms, hyperpolarization‐activated cyclic nucleotide‐gated channel, lateral habenula, neuronal excitability

## Abstract

**Aims:**

Comorbid anxiodepressive‐like symptoms (CADS) in chronic pain are closely related to the overactivation of the lateral habenula (LHb). Hyperpolarization‐activated cyclic nucleotide‐gated (HCN) channels have been implicated to play a key role in regulating neuronal excitability. However, the role of HCN channels in the LHb during CADS has not yet been characterized. This study aimed to investigate the effect of HCN channels in the LHb on CADS during chronic pain.

**Methods:**

After chronic neuropathic pain induction by spared nerve injury (SNI), mice underwent a sucrose preference test, forced swimming test, tail suspension test, open‐field test, and elevated plus maze test to evaluate their anxiodepressive‐like behaviors. Electrophysiological recordings, immunohistochemistry, Western blotting, pharmacological experiments, and virus knockdown strategies were used to investigate the underlying mechanisms.

**Results:**

Evident anxiodepressive‐like behaviors were observed 6w after the SNI surgery, accompanied by increased neuronal excitability, enhanced HCN channel function, and increased expression of HCN2 isoforms in the LHb. Either pharmacological inhibition or virus knockdown of HCN2 channels significantly reduced LHb neuronal excitability and ameliorated both pain and depressive‐like behaviors.

**Conclusion:**

Our results indicated that the LHb neurons were hyperactive under CADS in chronic pain, and this hyperactivation possibly resulted from the enhanced function of HCN channels and up‐regulation of HCN2 isoforms.

## Graphical abstract

Increased LHb neuronal excitability, which potentially resulted from enhanced function of HCN channels and increased expression of HCN2 isoforms, is crucial for the development of CADS in chronic pain. Either bilateral microinjection of ZD7288 into the LHb to inhibit HCN channels or the specific knockdown of HCN2 channels could obviously decrease the LHb neuronal excitability and produce significant analgesic and antidepressant effects.

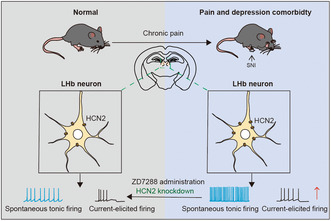

## 1 INTRODUCTION

Patients with long‐lasting or recurrent chronic pain are prone to adverse psychological consequences, particularly major depression and anxiety.^1^, ^2^, ^3^ The comorbidity of chronic pain and psychological disorders—a common clinical problem—poses significant challenges for current effective antidepressant and analgesic therapies, which are characterized by a lack of precise intervention targets and unavoidable severe side effects.^4^, ^5^, ^6^ Despite a rapidly growing body of literature focusing on the pathogenesis of the comorbidity of chronic pain and psychological disorders at the neural circuit level,^7^, ^8^, ^9^, ^10^, ^11^, ^12^, ^13^ further investigations are required to elucidate its underlying neural molecular mechanisms.

Lateral habenula (LHb), a nucleus of the epithalamus primarily comprised of glutamatergic (Glu) neurons,^14^ has been considered as the “anti‐reward center” because its malfunction is closely associated with psychological disorders, particularly major depression.^15^, ^16^ Numerous studies have consistently observed significant overactivation of the LHb, no matter under major depression or chronic pain.^17^, ^18^, ^19^, ^20^ Various techniques to suppress the LHb's activity can produce significant antidepressant and analgesic effects.^21^, ^22^, ^23^, ^24^, ^25^ Noteworthily, recent studies have confirmed enhanced neuronal excitability and potentiated synaptic activity within the LHb during chronic pain and major depression comorbidity, suggesting that the LHb may serve as a convergent brain region in both chronic pain and major depression.^26^, ^27^

It is well known that the regulation of neuronal excitability relies on the expression and function of a range of ion channels within the nervous system. Hyperpolarization‐activated cyclic nucleotide‐gated (HCN) channel—considered a pacemaker channel—can be activated when the membrane potential hyperpolarizes below −50 mV and mediates a depolarizing mixed Na^+^ and K^+^ current which is termed as Ih.^28^ Further, HCN channels comprise four subunits (HCN1‐4), which are widely expressed in both the peripheral and central nervous systems and closely related to the regulation of neuronal excitability and neurotransmitter release.^29^, ^30^ Notably, a recent study^31^ reported increased expression of HCN1 and HCN2 in the thalamus and hippocampus under the comorbidity of chronic pain and depression. Previous studies have indicated that four subunits of the HCN channel were enriched in the LHb at the RNA level.^32^, ^33^ However, what specific role do HCN channels of the LHb play under CADS in chronic pain remains unknown.

To address these gaps, we employed electrophysiology, immunohistochemical techniques, Western blot (WB) analysis, pharmacological interventions, and virus knockdown strategies, and found that LHb neuronal excitability was significantly increased under CADS in chronic neuropathic pain induced by spared nerve injury (SNI). Additionally, we systematically elucidated that HCN channel function was enhanced and that HCN2 isoform expression was increased in mice exhibiting CADS. Notably, either bilateral microinjection of ZD7288 into the LHb to inhibit HCN channels or the specific knockdown of HCN2 channels produced conspicuous improvement in pain and comorbid depressive‐like behaviors. These findings in this study provided both cellular and functional support for the pivotal role played by HCN channels, particularly HCN2 isoforms in the LHb, in the comorbidity of chronic neuropathic pain and depression.

## 2 MATERIALS AND METHODS

### 2.1 Animals

Male wild‐type C57BL/6J mice (8–12 weeks of age, 20–30 g) were used for all experiments. The mice were housed in controlled conditions with a temperature range of 23–25°C and humidity maintained at 40%–60%, with free access to food and water ad libitum under a 12‐h light/dark cycle (lights on from 7 a.m. to 7 p.m.). The mice were kept in groups of 4–5 per cage and were randomly assigned to each experimental group. All experimental procedures were performed in strict accordance with the International Association for the Study of Pain Guidelines and approved by the Institutional Animal Care and Use Committee of the First Affiliated Hospital of Nanchang University (CDYFY‐IACUC‐202208QR002). Optimal efforts were invested to minimize animal suffering and reduce the number of animals used in the experiments.

### 2.2 Animal models of chronic neuropathic pain

Spared nerve injury (SNI) was used to develop chronic neuropathic pain. Following previously described surgical procedures,^34^ under isoflurane anesthesia, the left femoral segment's skin was aseptically dissected, and the thigh muscles were bluntly separated to expose the sciatic nerve comprising the common peroneal, tibial nerve, and sural nerves. The tibial and common peroneal nerves were ligated with nonabsorbent 7–0 sutures and then cut. Subsequently, 1–2 mm sections from the ligated site were additionally removed to prevent nerve regeneration. The sural nerve was left intact. After the operation, the muscles and skin were sutured layer by layer. The Sham group underwent the same procedures as the SNI group, except that the sciatic nerve was left intact. Mechanical pain thresholds were evaluated preoperatively and at 1, 3, 7, 14, 21, 28, 35, and 42 days postoperatively.

### 2.3 Behavioral assays

Behavioral assays—except for the sucrose preference test—were performed during the light phase. Before the test, mice were transferred to the testing room for at least 24 h to habituate. A video tracking system was used to record the various behaviors of the mice, and subsequent data analysis was performed by experimenters who were blinded to the group identities. Noteworthily, if mice were enrolled in the tail suspension test (TST), they would no longer participate in the forced swimming test (FST), and vice versa.

#### 2.3.1 von Frey test

The von Frey test was used to assess the mechanical pain thresholds of mice. The mice were placed in plexiglass chambers (10 × 10 × 13 cm) with a metal mesh grid at the bottom and positioned 30 cm away from the table. After a minimum of 30 min of adaptation, the paw withdrawal threshold (PWT) in response to mechanical stimuli was assessed using a set of von Frey filaments with logarithmically increasing stiffness (0.02–2.56 g, North Coast Stoelting, USA). The filaments were applied perpendicularly to the skin of the left hind paws' lateral plantar area (innervated by the sural nerve). Positive responses were recorded when the mouse exhibited quick withdrawal, licking, or shaking upon stimulation. The 50% PWT was determined using the up–down method, as described in a previous study.^35^

#### 2.3.2 Sucrose preference test (SPT)

The mice were individually housed under a 12‐h light/dark cycle (lights on from 7 a.m. to 7 p.m.). During the adaptation phase, two bottles of water were provided to each cage for 48 h, followed by two bottles of 2% sucrose for an additional 48 h. Thereafter, the positions of the two bottles were switched every 12 h to avoid position preference. Subsequently, the two bottles of 2% sucrose were removed for 24 h, with free access to food ad libitum. The testing phase commenced by providing the mice with one bottle of water and one bottle of 2% sucrose solution for 2 h during the dark phase. After 1 h, the positions of the two bottles were interchanged to eliminate position preferences. Water and sucrose consumption were measured, and the sucrose preference value was calculated by dividing the amount of sucrose consumed by the total consumption of both water and sucrose.^21^

#### 2.3.3 Forced swimming test (FST)

The mice were placed in a transparent glass cylinder (with 20 cm height and 15 cm diameter) filled with water (23–25°C), with a depth of 10–15 cm to prevent mice from touching the bottom with their tails or hind limbs. They were allowed to swim freely for 6 min under normal light. The water was replaced in each subsequent experiment. The immobility time during the last 4 min was calculated, and immobility was defined as mice floating or making slight movements to maintain balance in the water.^21^

#### 2.3.4 Tailed suspension test (TST)

Using tape to secure the tails, the mice were individually suspended upside down, approximately 50 cm above the surface of a table. A transparent cylinder was placed on their tails to prevent climbing. The entire test lasted for 6 min, during which the immobility time for the last 5 min was recorded. Immobility was defined as remaining still or passively swaying because of inertia.^27^

#### 2.3.5 Open‐field test (OFT)

The mice were placed at the center of an open‐field apparatus (50 × 50 × 25 cm). The center area was defined as a 20 × 20 cm square area. The mice were allowed to explore the entire area freely for 30 min. The total distance traveled in 30 min was used to evaluate general locomotor activity, and the time spent in the center area during the first 5 min was used to evaluate their anxiety‐like behavior. The box was cleaned by using 75% ethanol to remove any residual odor from the mice before conducting the next experiment.^27^ The videos were automatically analyzed using MATLAB R2018b (Math Works, MA, USA). The MATLAB source code can be obtained from http://www.seas.upenn.edu/~molneuro/autotyping.

#### 2.3.6 Elevated plus maze test (EPM)

The EPM test's setup comprises two open arms (30 × 5 cm), two closed arms (30 × 5 × 20 cm), and a central platform area (5 × 5 cm) elevated above the ground. The mice were placed in the central area—facing one of the closed arms—and allowed to explore the maze freely for 5 min. The amount of time spent in and the number of open‐arm entries were analyzed to evaluate their anxiety‐like behavior. The area was cleaned by using 75% ethanol to prepare for the next test.^27^ The videos were analyzed using Visu Track.lnk software (XR‐VT, Shanghai Xinruan Information Technology Co., Ltd., China).

### 2.4 Western blot (WB)

Following cardiac perfusion with cold saline, the LHb tissues were rapidly dissected from 300 μm thick brain slices which were derived from a vibratome (VT1000S, Leica, Germany) and immediately frozen by liquid nitrogen. Thereafter, the LHb tissues were homogenized in RIPA buffer (R0010, Solarbio, China) containing a mixture of protease inhibitor (P8340, Sigma, USA) on ice using an ultrasonic lysis apparatus (JY92‐IIN, Ningbo Scientz Biotechnology Co., Ltd., China). Subsequently, the homogenates were then subjected to centrifugation at 12,000 rpm for 15 min at 4°C. For WB assays, protein samples (60 μg protein for HCN and β‐actin) were loaded into each well—separated through sodium dodecylsulfate (SDS)‐polyacrylamide gel electrophoresis (PAGE) (P1200, Solarbio, China) and, subsequently, transferred onto preactivated polyvinylidene fluoride membranes (IPVH00010, Millipore, USA) using methanol. The membranes were blocked with 5% nonfat milk (232,100, Bio‐Rad, USA) in TBST for 1 h at room temperature. Thereafter, the membranes were incubated with rabbit polyclonal anti‐HCN1 (1:1000; Proteintech, USA), HCN2 (1:1000; proteintech, USA), HCN3 (1:500; Alomone Labs, Israel), HCN4 (1:500; Alomone Labs, Israel), and rabbit polyclonal anti‐β‐actin (1:1000; Proteintech, USA) antibodies overnight at 4°C. Following three washes with TBST (10 min each), the membranes were incubated with goat anti‐rabbit IgG secondary antibody (1:1000; Thermo Fisher Scientific, USA) for 1 h at room temperature. After another three washes, the target protein bands were detected using a high‐sensitivity ECL reagent (B500023, Proteintech, USA) and an imaging system (iBright FL1000, Thermo Fisher Scientific, USA). The intensities of all bands were quantified using Image J software (NIH Image analysis website: http://rsb.info.nih.gov/ij/).

### 2.5 Immunohistochemistry (IHC)

The mice were deeply anesthetized with urethane (1.5 g/kg, i.p.) and perfused intracardially with cold saline, followed by cold 4% paraformaldehyde (PFA) in 0.1 M phosphate buffer. For Fos protein labeling, mice were sacrificed and perfused 1 h after the last behavioral test. Subsequently, the brains were removed and post‐fixed in 4% PFA for 6 h at 4°C, and then cryoprotected in 30% sucrose solution for 3 days. The brains were embedded in tissue freezing medium at −20°C, and coronal sections (30 μm) were prepared by a frozen microtome (CM1950, Leica, Germany) and immersed in phosphate‐buffered saline (PBS) for further immunostaining. All sections were rinsed using PBS and incubated in a blocking solution containing 0.3% Trion X‐100 (X‐100, Sigma, USA), 1% bovine serum albumin (A4503, Sigma, USA), and 1% normal donkey serum (ab7475, Sigma, USA). Subsequently, the sections were incubated with primary antibodies including anti‐HCN1 (55222‐1‐AP, 1:200, rabbit, Proteintech, USA), anti‐HCN2 (55245‐1‐AP, 1:200, rabbit, Proteintech, USA), anti‐HCN3 (APC‐057, 1:200, rabbit, Alomone Labs, Israel), anti‐HCN4 (APC‐052, 1:200, rabbit, Alomone Labs, Israel), anti‐NeuN (26004, 1:200, Guinea pig, Synaptic Systems, Germany), anti‐GFAP (ab53554, 1:400, Goat, Abcam, UK), anti‐Iba1 (011‐27991, 1:200, Goat, Wako, Japan), and anti‐c‐FOS (2250S, 1:1000, Rabbit, Cell Signaling Technology, USA) for 3 days at 4°C. Subsequently, the sections were rinsed with PBS three times and incubated with the appropriate secondary antibodies, namely, Alexa Fluor 555 (A31572, 1: 400, Rabbit, Invitrogen, USA), Alexa Fluor 555 (A21432, 1: 400, Goat, Invitrogen, USA), Alexa Fluor 647 (A31573, 1: 400, Rabbit, Invitrogen, USA), or Cy5 (706‐175‐148, 1:400, guinea pig, Jackson ImmunoResearch, USA) for 1 h at room temperature. After washing them three times, brain sections were fixed with anti‐fluorescence‐attenuating tablets (0100‐20, Southern Biotech, USA). Thereafter, the fluorescence signals were then observed under a Zeiss LSM700 confocal microscope using ZEN 2010 software and Leica Stellaris 5 confocal microscope using LAS X.

### 2.6 Virus injection

Under isoflurane anesthesia, the mice were placed in a stereotaxic frame (RWD Life Science). We targeted the LHb with stereotaxic coordinates ranging from bregma in mm (anterior–posterior (AP): −1.65; medial‐lateral (ML): ±0.46; dorsal‐ventral (DV): −2.7) according to the mouse brain atlas. A total of 40 nL pAAV‐*U6*‐shRNA‐scramble‐*CMV‐eGFP‐3Flag* (AAV‐shRNA‐scramble‐*GFP*) or pAAV‐U6‐shRNA‐*Hcn2‐CMV‐eGFP‐3Flag* (AAV‐shRNA‐*Hcn2‐GFP*) (Genechem, Shanghai, China) were bilaterally injected into the LHb using a fine glass electrode. The glass electrode was left in place for 10 min before withdrawal. All mice that received the virus injection were kept at least 3 weeks after brain injection for virus expression and further experiments.

### 2.7 Brain slice preparation

After the last behavioral test, the mice were deeply anesthetized with urethane (1.5 g/kg, i.p.) and intracardially perfused with an ice‐cold sucrose‐based artificial cerebrospinal fluid (s‐ACSF)—preoxygenated with 95% O_2_ + 5% CO_2_, containing the following (in mM): 204 sucrose, 2.5 KCl, 3.5 MgCl_2_, 0.5 CaCl_2_, 1.25 NaH_2_PO_4_, 0.4 ascorbic acid, 2 sodium pyruvate, 11 D‐glucose, 25 NaHCO_3_, and 1 kynurenic acid. Subsequently, the brain was rapidly removed and placed in the same solution. Coronal slices containing the habenula (280 μm) were sectioned into ice‐cold s‐ACSF using a vibrating microtome (VT1000S, Leica, Germany). Subsequently, the brain slices were transferred to standard ACSF (preoxygenated with 95% O_2_ + 5%CO_2_) at 32°C to incubate for 30 min. Thereafter, the slices were allowed to recover at room temperature for at least 30 min before the electrophysiological recording. The standard ACSF consists of (in mM) 117 NaCl, 3.6 KCl, 2.5 CaCl_2_, 1.2 MgCl_2_, 1.2 NaH_2_PO_4_, 25 NaHCO_3_, 11 D‐glucose, and 2 sodium pyruvic acid.

### 2.8 In vitro electrophysiological recordings

Brain slices containing the LHb were transferred into the recording chamber and perfused continuously with standard ACSF (2–4 mL/min) at room temperature (23–25°C). Further, LHb neurons were visualized using an Olympus microscope (BX51WI, Olympus Corp., Japan) and an IR‐DIC camera (IR‐1000, Dage, USA), and GFP‐positive LHb neurons were identified using a 40X objective with blue light (470 nm; M470L4‐C1 or M590L4‐C1, Thorlabs Inc., NJ, USA). Patch pipettes (3–6 MΩ) were pulled from borosilicate glass capillaries (1.5 mm OD, 1.12 mm ID, World Precision Instruments, USA) using a Sutter P‐97 puller (Sutter Instruments, Novato, USA). The intracellular solution contained (in mM) 130 K‐gluconate, 5 KCl, 10 Na_2_‐phosphocreatine, 0.5 EGTA, 10 HEPES, 4 Mg‐ATP, and 0.3 Li‐GTP (pH 7.3 adjusted with KOH, 295 mOsm). Patch‐clamp recordings in the whole‐cell configuration were performed using an EPC‐10 amplifier and Patchmaster software (HEKA Electronics, Lambrecht, Germany).

The LHb neurons' resting membrane potential (RMP) was recorded at 0 pA within 20 s after whole‐cell configuration. Spontaneous neuronal activity was recorded under current clamp (*I* = 0 pA). LHb neurons showed three spontaneous firing patterns at 0 pA. Silent neurons showed no spontaneous spikes during recording. Tonic‐firing neurons showed spontaneous tonic trains of action potentials (APs) at frequency no less than 0.1 Hz. Burst‐firing neurons showed spontaneous clusters of spikes with an initially high but progressively declining intra‐burst firing frequency in each burst and the minimum number of spikes in a burst was set at 2. The input resistance (Rin) was measured at −70 mV using a current change induced by a −10 mV voltage pulse. To record the LHb neurons' AP firing, a series of 1 s current pulses from 0 to 100 pA with 20 pA steps were applied to evoke spikes. Only neurons exhibiting overshooting APs were included for further analysis.

Under voltage‐clamp conditions, evoked Ih currents were recorded by applying a series of hyperpolarizing voltage steps (from −60 mV to −130 mV in increments of −10 mV) for a duration of 1000 ms, beginning from a holding potential of –50 mV. The amplitude of Ih was measured as the difference between the initial inward current and the steady state current at −130 mV, unless mentioned otherwise. To determine the half‐activation potential (*V*_1/2_) of Ih, neurons were clamped at −50 mV under voltage clamp, subjected to a series of voltage stimuli from −60 mV to −130 mV, and subsequently clamped at −130 mV. The activation curve of Ih was fitted using the Boltzmann equation, *I*/*I*_max_ = 1/ (1+ exp [(*V*_1/2_–*V*)/*k*)], where *I*_max_ is the maximal current amplitude, *V*_1/2_ is the midpoint potential, *V* is the membrane potential, and *k* is the slope factor. The Ih density was calculated by dividing the Ih amplitude by the neuronal capacitance.^36^

To examine drug effects on the LHb neuronal excitability, the LHb neurons were recorded for 5 min as a baseline and, subsequently, perfused with ZD7288 (10 μM) for 15 min.^37^

Neurons with RMP values more positive than −40 mV were excluded from the analysis. The series resistance typically ranged from 10 to 30 MΩ, and any neurons exhibiting a change in series resistance exceeding 20% were also excluded. Data were analyzed using Clampfit 10.7 (Molecular Devices, USA).

### 2.9 Cannula infusion experiment

A double‐guide cannula with a side‐by‐side distance of 1.0 mm (plastics one) was bilaterally implanted over the LHb (−1.65 mm AP; ± 0.46 mm ML; −2.20 mm DV) of the C57BL/6J mice. A double dummy cannula with a 0.5 mm extension beyond the tip of the guide cannula—secured with a dust cap—was inserted into the guide cannula to avoid clogging during the recovery period.^38^ After a recovery period of at least 7 days, ZD7288 (10 μg/kg, Sigma)^31^, ^39^ dissolved in 0.9% saline (NS) was infused into each side of the LHb using a Hamilton micro‐syringe connected to a double injector cannula that was via a polyethylene pipe filled with mineral oil. A total volume of 200 nL of ZD7288 was infused at a rate of 100 nL/min. To avoid spreading the drug along the injection track, the injector cannula remained in the LHb for an additional 10 min. Mechanical allodynia of the mice was assessed at 30, 60, and 90 min after drug administration, while the OFT, EPM, TST, and FST were performed 60 min after drug infusion.

### 2.10 Statistical analysis

Statistical analyses were performed using GraphPad Prism (version 8.0, GraphPad Software, San Diego, CA, USA). Data are presented as mean ± SEM or Min to Max. Shapiro–Wilk test and Brown‐Forsythe tests were used to assess the normality and the homogeneity of variances, respectively. If normality and the homogeneity of variance between groups were achieved, a *t*‐test or one‐way ANOVA (followed by Fisher's LSD multiple comparisons test) was employed. In cases where normality and the homogeneity of variance were not fulfilled, the Mann–Whitney test, Kruskal–Wallis one‐way ANOVA test (followed by Dunn's test), or Wilcoxon paired test was conducted. Two‐way ANOVA (followed by Fisher's LSD multiple comparisons test) or mixed‐effects analysis was applied when appropriate. The Chi‐square test was conducted to determine differences in the proportion of three firing types of LHb neurons, as well as the proportion of LHb neurons with and without Ih, between Sham and SNI groups. Statistical significance was set at *p* < 0.05. The statistical analysis and *n* numbers contained in all figures and supplementary figures could be seen in Table S1.

## 3 RESULTS

### 3.1 Multiple anxiodepressive‐like behaviors 6w after SNI surgery

**FIGURE 1:**
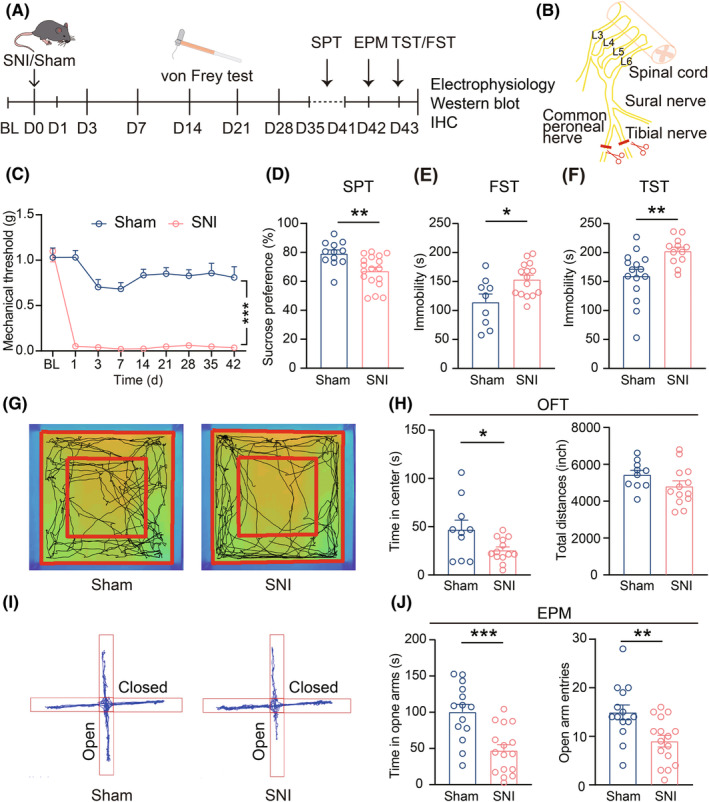
Multiple anxiodepressive‐like behaviors are observed in SNI 6w mice. (A) Schematic diagram of the experimental process. (B) Schematic for inducing neuropathic pain through the SNI surgery. (C) Time course of SNI‐induced changes in mechanical pain threshold measured by von Frey test (Sham, *n* = 16; SNI, *n* = 16, *F*_(4.158,62.36)_ = 14.70, *p* < 0.0001, Two‐way ANOVA). (D) Performance of Sham or SNI 6w mice in SPT (Sham, *n* = 12; SNI, *n* = 18, *p* = 0.0032, unpaired *t*‐test). (E) Immobility time of Sham or SNI 6w mice in FST (Sham, *n* = 9; SNI, *n* = 15, *p* = 0.0116, unpaired *t*‐test). (F) Immobility time of Sham or SNI 6w mice in TST (Sham, *n* = 15; SNI, *n* = 12, *p* = 0.0055, unpaired *t*‐test). (G) Representative images of activity tracking of Sham or SNI 6w mice in OFT. (H) Left: Time spent in center region within 5 min in OFT (Sham, *n* = 13; SNI, *n* = 13, *p* = 0.0323, unpaired *t*‐test). Right: Total distance traveled over a 30 min period in OFT (Sham, *n* = 13; SNI, *n* = 13, *p* = 0.1304, unpaired *t*‐test). (I) Representative movement tracks of Sham or SNI 6w mice in EPM within 5 min. (J) Left: Time spent in open arms in EPM (Sham, *n* = 13; SNI, *n* = 16, *p* = 0.0003, unpaired *t*‐test). Right: Open arm entries in EPM (Sham, *n* = 13; SNI, *n* = 16, *p* = 0.0085, unpaired *t*‐test). Data are shown as mean ± SEM. *p < 0.05, **p < 0.01, ***p < 0.001.

To explore the neural mechanisms underlying CADS in chronic pain, we utilized the SNI model to induce chronic neuropathic pain based on a previous study^27^ (Figure 1A,B). von Frey tests revealed that compared to the Sham group, the PWT of SNI mice was significantly reduced from day 1 post‐surgery and remained consistently lower for at least 42 days throughout the experimental period (Figure 1C). These findings suggested that SNI surgery can induce stable and persistent chronic neuropathic pain. To assess anxiodepressive‐like behaviors in mice, a series of behavioral assays—including the SPT, FST, TST, OFT, and EPM—were performed at 6 weeks after SNI surgery (SNI 6w). Compared with the Sham group, SNI 6w mice exhibited a significant decrease in sucrose preference value in the SPT (Figure 1D), along with an increase in immobility time in both the FST (Figure 1E) and TST (Figure 1F), indicating that SNI 6w mice exhibited anhedonia and despair—the core symptoms of major depression. Additionally, SNI 6w mice spent less time in the center zone in the OFT (Figure 1G,H) and had fewer open‐arm entries in the EPM (Figure 1I,J), indicating noticeable anxiety‐like behaviors. Noteworthily, the locomotion abilities of mice remained unaffected by SNI surgery, as evidenced by the lack of significant difference in the total distance traveled during a 30 min observation period in the OFT between these two groups (Figure 1H). Based on these results, we confirmed that mice exhibited various multiple anxiodepressive‐like behaviors 6w post the SNI surgery.

### 3.2 Activity of the LHb neurons enhanced in SNI 6w mice

**FIGURE 2:**
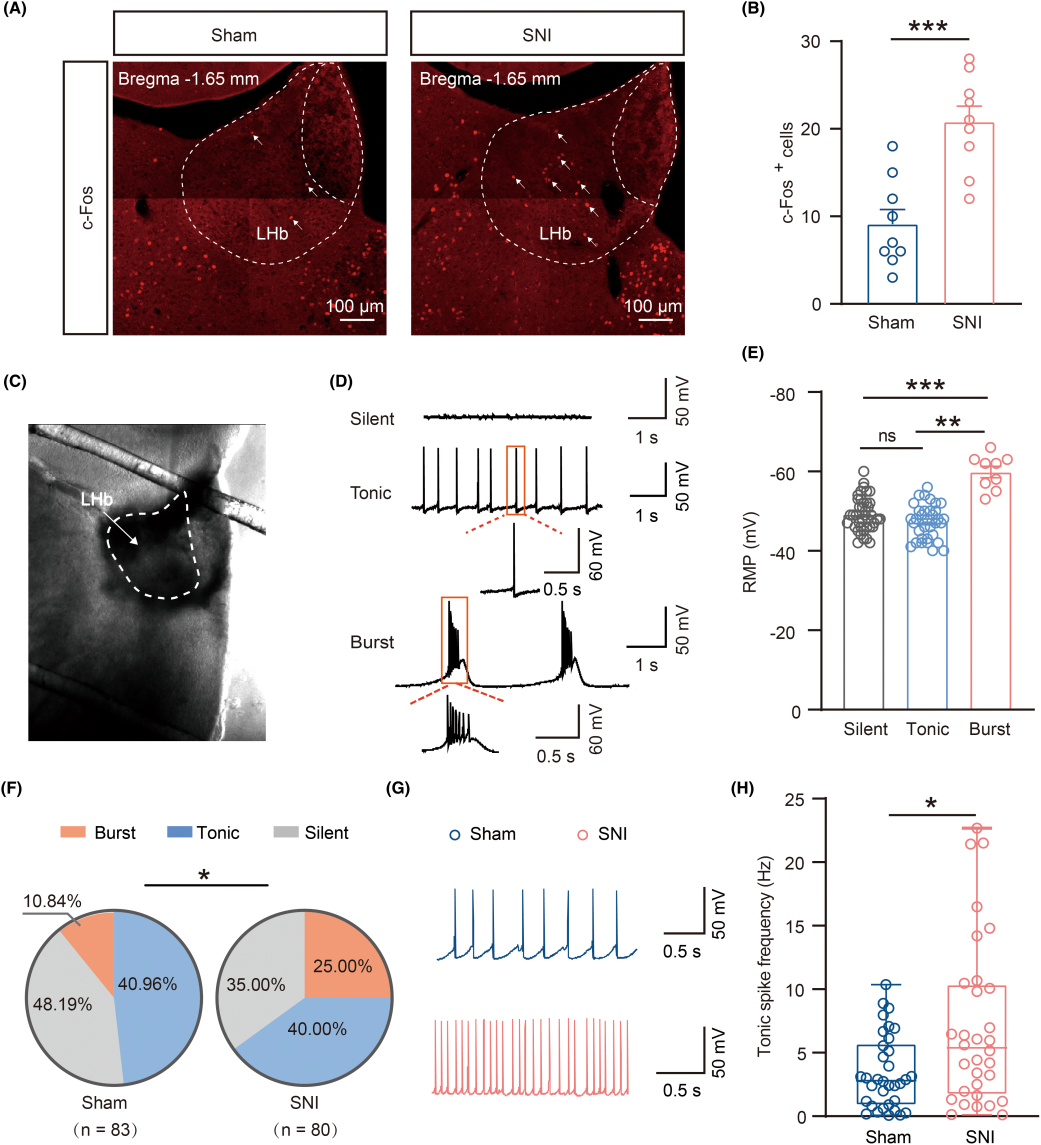
Increased neuronal excitability of the LHb neurons in SNI 6w mice. (A) Representative images of c‐FOS expression in LHb of Sham and SNI 6w mice. Scale bar, 100 μm. White arrows indicate c‐FOS expression in LHb. (B) Total number of c‐FOS‐positive neurons in LHb of Sham and SNI 6w mice (*n* = 9 brain sections from 3 mice for each group, *p* = 0.0002, unpaired *t*‐test). (C) Representative figure of LHb under the IR‐DIC camera. (D) Representative traces of spontaneous activity of silent, tonic‐firing, and burst‐firing in LHb. (E) The RMP of three firing types of the LHb neurons (*n* = 9 burst neurons, 34 tonic neurons, 40 silent neurons from 28 mice; *F*_(2,80)_ = 30.56, silent vs burst: *p* < 0.0001, tonic vs burst: *p* < 0.0001, One‐way ANOVA). (F) Pie charts showing the percentage of three types of LHb neurons. (Sham, *n* = 9 burst neurons, 34 tonic neurons, and 40 silent neurons from 28 mice; SNI, *n* = 20 burst neurons, 32 tonic neurons, and 28 silent neurons from 25 mice, *p* = 0.0429, Chi‐square test). (G, H) Representative traces (G) and statistical data (H) of tonic firing of LHb neurons in Sham and SNI 6w mice (Sham, *n* = 34 neurons from 19 mice; SNI, *n* = 32 neurons from 10 mice, *p* = 0.0211, Mann–Whitney test). Data are shown as mean ± SEM or Min to Max. *p < 0.05, **p < 0.01, ***p < 0.001.

**FIGURE 3:**
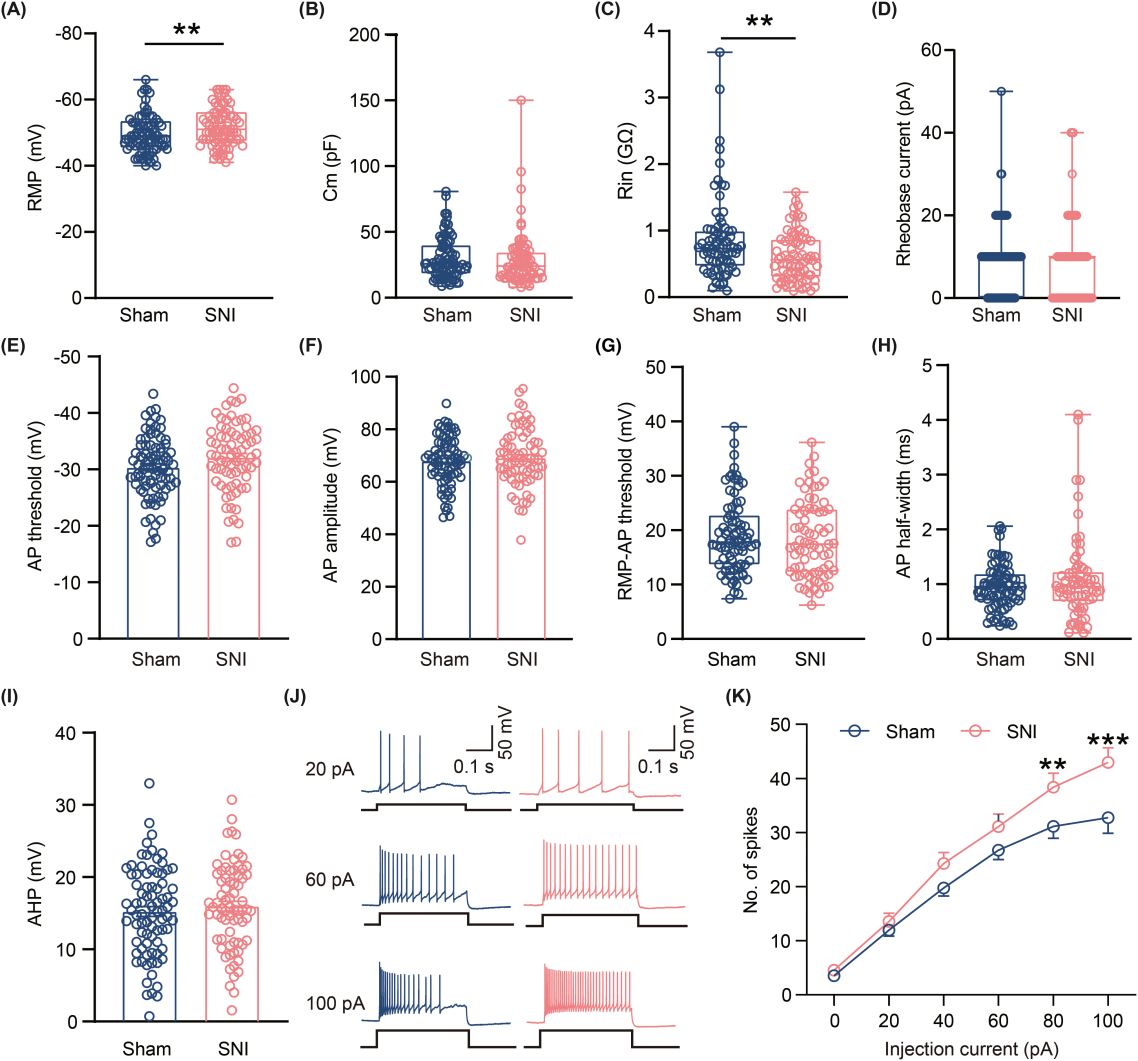
Increased intrinsic excitability of the LHb neurons in SNI 6w mice. (A–D) Summary of the passive membrane properties of LHb neurons in Sham and SNI 6w mice (Sham, *n* = 81–83 neurons from 25 to 28 mice; SNI, *n* = 73–80 neurons from 22 to 25 mice). (A) RMP (*p* = 0.0098, Mann–Whitney test); (B) Cm (*p* = 0.2685, Mann–Whitney test); (C) Rin (*p* = 0.0072, Mann–Whitney test); (D) Rheobase current (*p* = 0.1631, Mann–Whitney test). (E–I), Summary of the active membrane properties of LHb neurons in Sham and SNI 6w mice (Sham, *n* = 81 neurons from 29 mice; SNI, *n* = 73 neurons from 26 mice); (E) AP threshold (*p* = 0.0587, unpaired *t*‐test); (F) AP amplitude (*p* = 0.4393, unpaired *t*‐test); (G) RMP‐AP threshold (*p* = 0.4843, Mann–Whitney test); (H) AP half‐width (*p* = 0.8083, Mann–Whitney test); (I) AHP (*p* = 0.4571, unpaired *t*‐test). (J) Representative traces of the spike of LHb neurons responded to 20 pA, 60 pA and 100 pA in Sham and SNI 6w mice. (K) Frequency‐current (F–I) curves showing the number of spikes of LHb neurons responded to a series of 1 s current pulses from 0 pA to 100 pA with 20 pA steps in Sham and SNI 6w mice (Sham, *n* = 44–49 neurons from 24 mice; SNI, *n* = 43–44 neurons from 16 mice, *F*_(5,449)_ = 5.177, *p*_(80 pA)_ = 0.0084, *p*_(100 pA)_ = 0.0003, two‐way ANOVA). Data are shown as mean ± SEM or Min to Max. **p < 0.01, ***p < 0.001.

Extensive research conducted in animal and clinic settings has consistently emphasized the pivotal role of the hyperactive LHb neurons in the pathophysiology of major depression^21^, ^40^ as well as pain perception.^24^ Therefore, we first employed immunohistochemistry to explore the expression of c‐Fos—a reliable marker of immediate‐early gene expression—to detect alterations in LHb neurons' activity under CADS in chronic pain. Compared with Sham mice, a significant increase in c‐Fos‐positive neurons was observed in the LHb of SNI 6w mice (Figure 2A,B). To further elucidate the changes in LHb neuronal excitability, we performed whole‐cell patch‐clamp (Figure 2C) on LHb neurons and recorded spontaneous neuronal activity under the current clamp. Consistent with previous research, our results indicated that the LHb neurons exhibited three distinct spontaneous activity patterns—namely, silent, tonic‐firing, and burst‐firing. Notably, bursting neurons exhibited more hyperpolarized RMP than silent or tonic‐firing neurons^21^, ^32^ (Figure 2D,E). Furthermore, the proportion of burst‐firing neurons in the LHb increased significantly from 10.84% in Sham controls to 25.00% in SNI 6w mice (Figure 2F). Additionally, the spike frequency of tonic‐firing neurons in the LHb was also obviously increased in SNI 6w mice as compared with Sham controls (Figure 2G,H). Subsequently, we tested several fundamental electrical properties of all LHb neurons, regardless of their spontaneous firing type, in SNI and Sham 6w mice. Although the membrane capacitance (Cm) and rheobase current remained unaltered in these two groups, a more hyperpolarized RMP and a significant decrease in Rin (Figure 3A–D) were observed in the LHb neurons of SNI 6w mice. While there were no significant differences observed in AP threshold (Figure 3E), AP amplitude (Figure 3F), RMP‐AP threshold (Figure 3G), AP half‐width (Figure 3H), and AHP (Figure 3I) between these two groups, the LHb neurons recorded in SNI 6w mice tended to fire more APs in response to a series of command current pulses (Figure 3J,K).

Furthermore, when dividing all recorded neurons into three types according to their spontaneous firing patterns, we identified that compared to Sham mice, the RMP of silent‐firing neurons was more hyperpolarized (Figure S1A), the Rin and rheobase current of both tonic‐firing neurons and silent‐firing neurons were smaller (Figure S1B), and the AP amplitude of silent‐firing neurons was higher (Figure S1E) in SNI 6w mice. All of the differences mentioned above were statistically significant. No significant differences were observed in the AP threshold (Figure S1D), RMP‐AP threshold (Figure S1F), AP half‐width (Figure S1G), and AHP (Figure S1H) between these two groups, irrespective of the type of firing neurons in the LHb. Collectively, these observations suggest that LHb neuronal excitability was abnormally enhanced in SNI 6w mice.

### 3.3 Four subtypes of HCN channels (HCN1‐4) were enriched in the LHb with distinct expression patterns and the LHb neurons with Ih exhibited higher neuronal excitability

**FIGURE 4:**
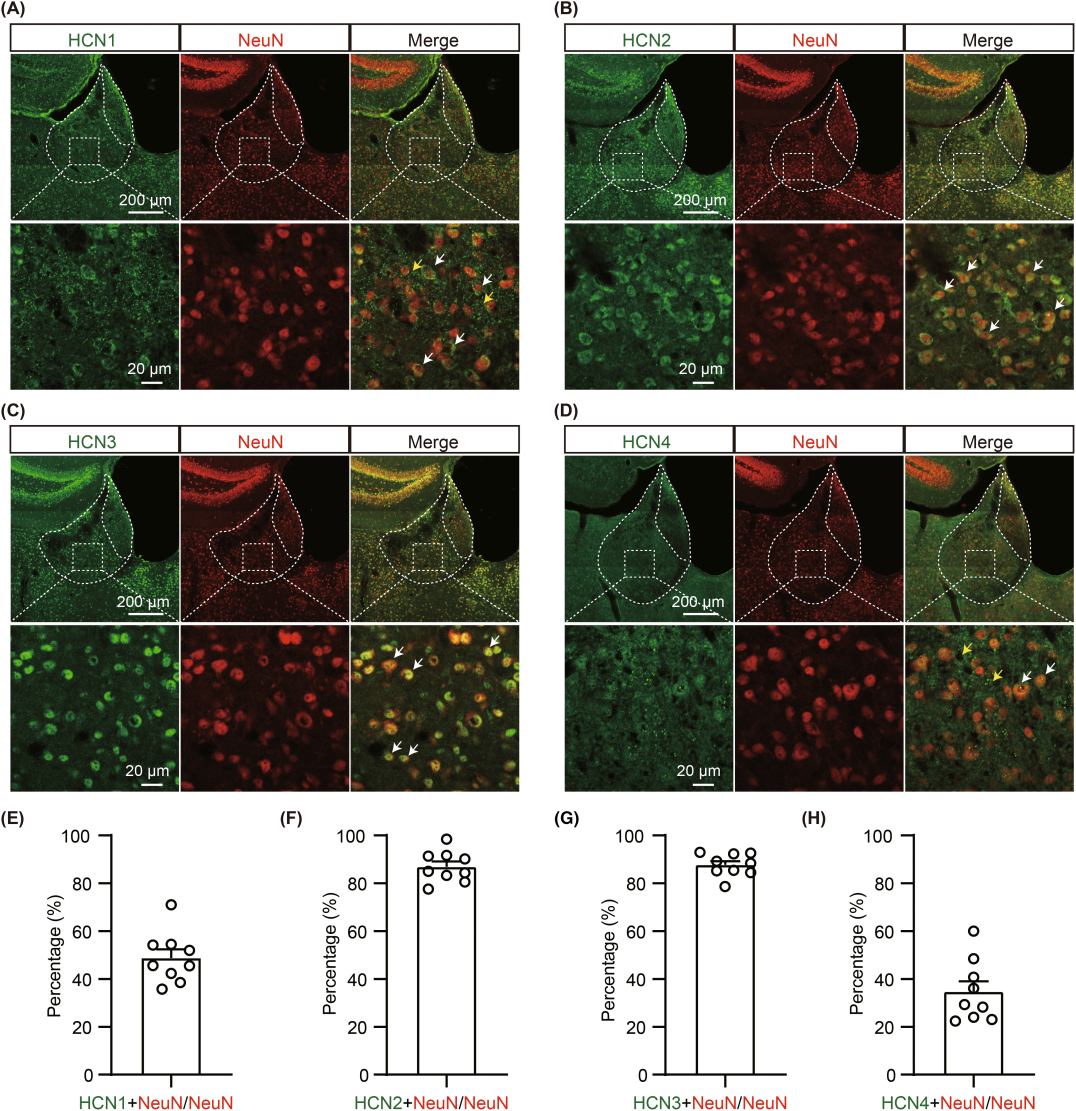
Four isoforms of HCN channels are enriched in the LHb. (A–D) Representative images of HCN channels (green), NeuN (red) and merged images of the HCN channel, and NeuN in LHb (*n* = 9 slices from 3 mice; Up, scale bar, 200 μm, Bottom, scale bar, 20 μm). White arrows indicate the HCN channel and soma co‐labeled neurons and yellow arrows indicate the HCN channel distributing on the non‐neuronal sites. (A) HCN1; (B) HCN2; (C) HCN3; (D) HCN4. (E–H) Percentage of co‐localized NeuN^+^ and HCN^+^ neurons (*n* = 9 slices from 3 mice). (E) HCN1 (48.82 ± 3.550%); (F) HCN2 (86.95 ± 2.160%); (G) HCN3 (87.71 ± 1.576%); (H) HCN4 (34.70 ± 4.315%). Data are shown as mean ± SEM.

We further explored the potential molecular mechanisms associated with LHb hyperexcitability under CADS in chronic neuropathic pain. Previous studies have demonstrated that HCN channels were abundantly expressed in the LHb at the RNA level and played a critical role in regulating neuronal properties including Rin, RMP, and firing activity.^32^, ^41^, ^42^, ^43^ To better understand their impact on cellular excitability of the LHb neurons, we first performed double‐immunolabeling experiments targeting HCN channel subunits (HCN1‐4) and NeuN (a neuronal marker). Immunoreactivity analysis revealed different expression patterns for all four HCN channel subunits in the LHb. Further, HCN2 and HCN3 exhibited predominant localization in the neuronal membrane or somata and displayed circular and globular staining patterns, while HCN1 and HCN4 were primarily distributed with punctate staining patterns (Figure 4A–D). Quantitative analysis indicated that HCN1 and HCN4 exhibited were weakly colocalized, whereas HCN2 and HCN3 exhibited a higher degree of co‐localization with NeuN (Figure 4E–H). We noticed that a minority of HCN channels didn't exhibit co‐localization with NeuN. To further explore whether there is co‐localization between HCN channels and glial cells, we performed double‐immunolabeling experiments targeting HCN channel subunits (HCN1‐4), GFAP (an astrocyte marker), and Iba1 (a microglia maker). Results showed that four isoforms of HCN channels were barely co‐localized with astrocyte and microglia (Figure S2).

**FIGURE 5:**
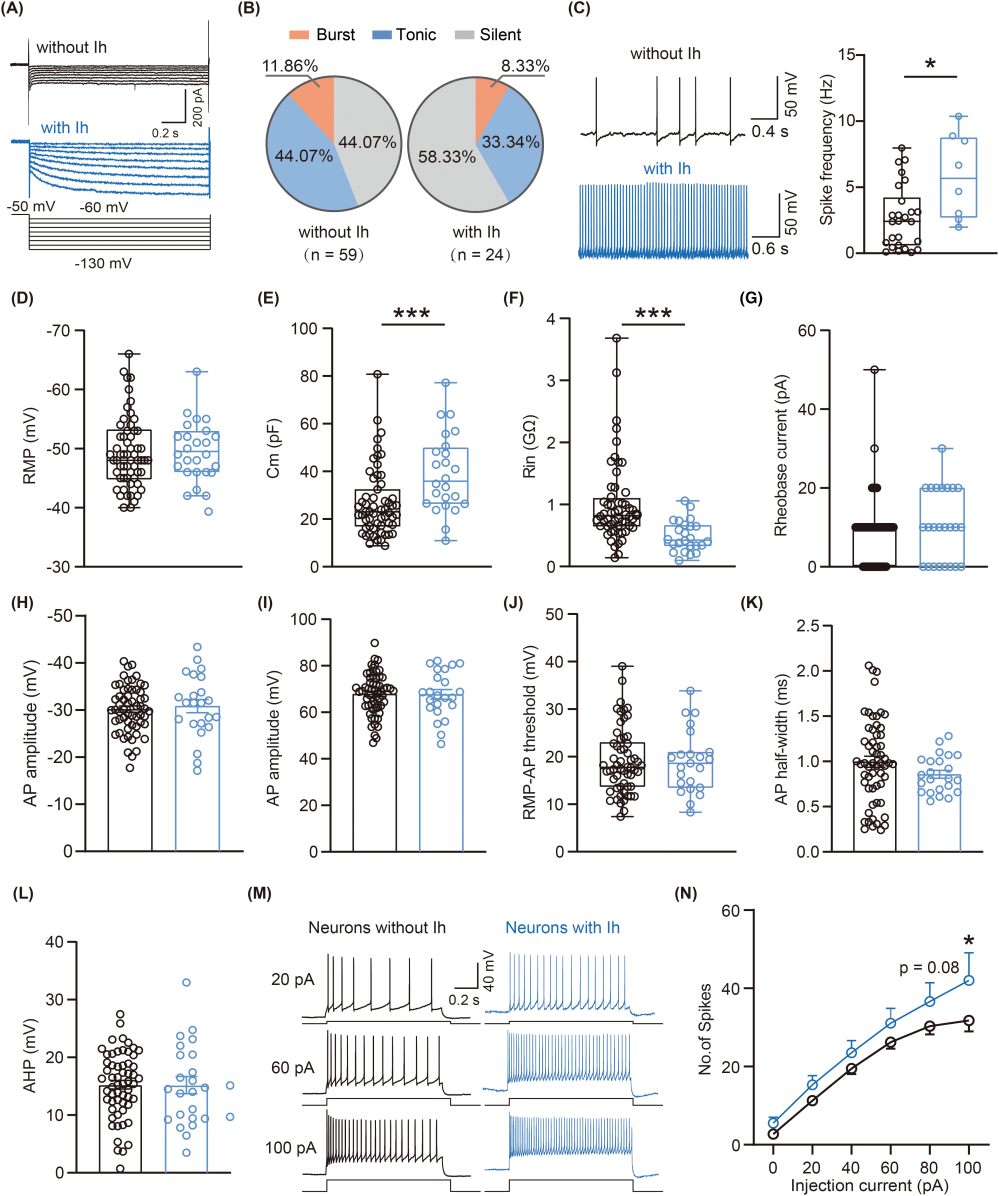
Neurons with Ih in the LHb of mice show higher neuronal excitability. (A) Representative traces of Ih in the LHb neurons. (B) Pie charts showing the percentage of three types of LHb neurons (without Ih, *n* = 7 burst neurons, 26 tonic neurons and 26 silent neurons from 19 mice; with Ih, *n* = 2 burst neurons, 8 tonic neurons, and 14 silent neurons from 12 mice, *p* = 0.4975, Chi‐square test). (C) Representative traces and statistical data of tonic firing of LHb neurons without and with Ih (without Ih, *n* = 26 neurons from 14 mice; with Ih, *n* = 8 neurons from 7 mice, *p* = 0.0150, Mann–Whitney test). (D–F) Summary of passive membrane properties of the LHb neurons without and with Ih (without Ih, *n* = 58–59 neurons from 19 mice; with Ih, *n* = 23–24 neurons from 14 mice). (D) RMP (*p* = 0.4276, Mann Whitney test); (E) Cm (*p* = 0.0003, Mann Whitney test); (F) Input resistance (*p* < 0.0001, Mann Whitney test). (G) Rheobase current (*p* = 0.2775, Mann Whitney test). (H–L) Summary of the active membrane properties of LHb neurons without and with Ih (without Ih, *n* = 58 neurons from 19 mice; with Ih, *n* = 23 neurons from 14 mice). (H) AP threshold (*p* = 0.6395, unpaired *t*‐test); (I) AP amplitude (*p* = 0.9578, unpaired *t*‐test); (J) RMP‐AP threshold (*p* = 0.9411, Mann Whitney test); (K) AP half‐width (*p* = 0.1723, unpaired *t*‐test); (L) AHP (*p* = 0.9649, unpaired *t*‐test). (M) Representative traces of spike without and with Ih responding to 20 pA, 60 pA and 100 pA. (N) Frequency‐current (F–I) curves show the number spikes of LHb neurons without and with Ih responding to a series of 1 s current pulses from 0 pA to 100 pA with 20 pA steps (without Ih, *n* = 27–30 neurons from 17 to 19 mice; with Ih, *n* = 12–16 mice from 12 to 14 mice, *F*_(5,213)_ = 0.8836, *p*_(80 pA)_ = 0.0811, *p*_(100 pA)_ = 0.0218, two‐way ANOVA). Data are shown as mean ± SEM or Min to Max. *p < 0.05, ***p < 0.001.

**FIGURE 6:**
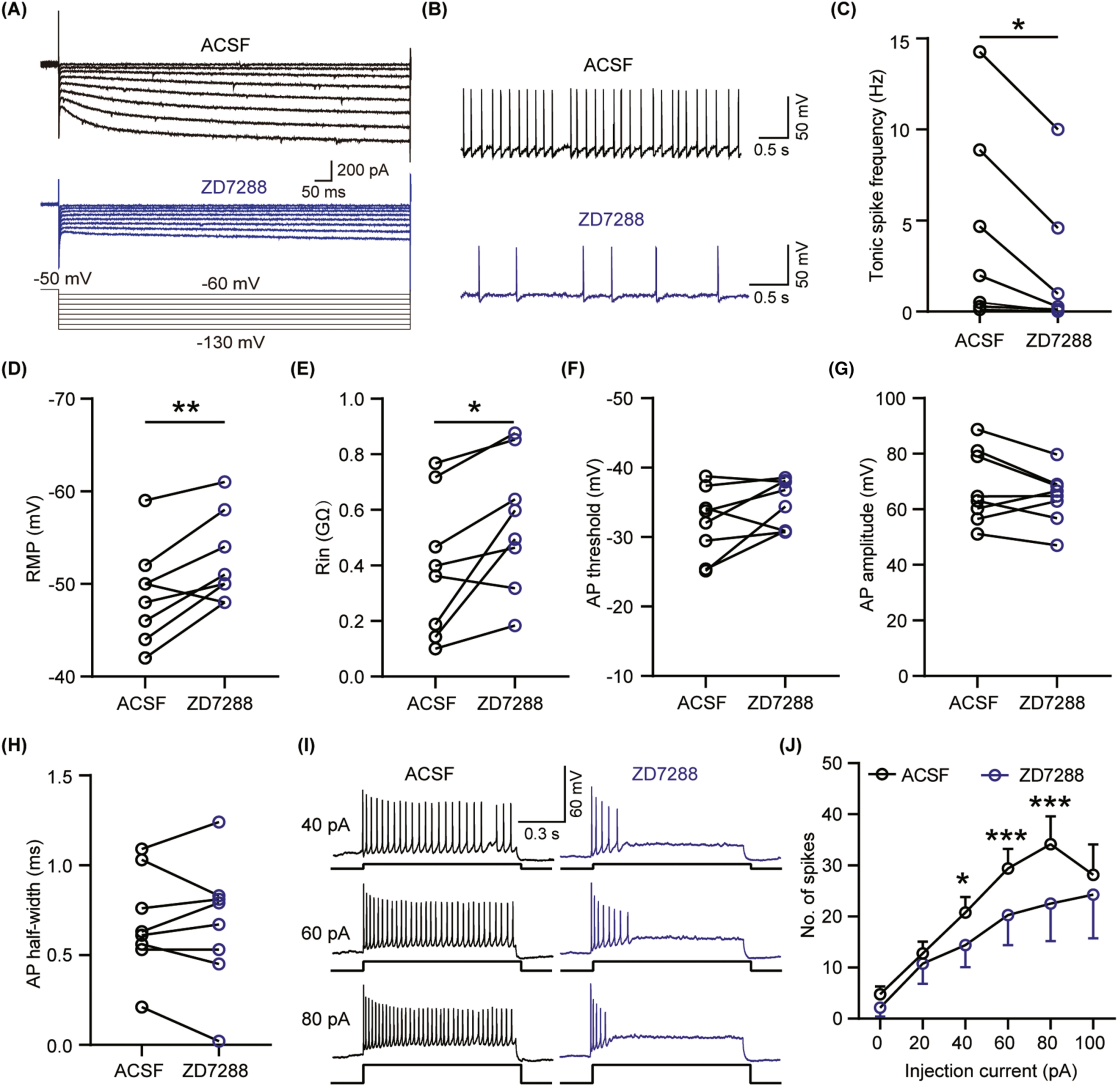
In vitro application of the HCN channel blocker decreases neuronal excitability of the LHb in mice. (A) Representative traces showing the effect of ZD7288 (10 μM) on the HCN current. (B, C) Representative traces (B) and statistics data (C) showing the effects of ZD7288 (10 μM) on tonic firing of LHb neurons in mice (*n* = 7 neurons from 6 mice for each group, *p* = 0.0156, paired *t*‐test). (D, E) Statistics data illustrating the effects of ZD7288 (10 μM) on some of the passive membrane properties of LHb neurons. (D) RMP (*n* = 6 neurons from 5 mice for each group, *p* = 0.0084, paired *t*‐test). (E) Rin (*n* = 8 neurons from 7 mice for each group, *p* = 0.0156, Wilcoxon test). (F–H) Statistics data showing the effects of ZD7288 (10 μM) on the active membrane properties of LHb neurons (*n* = 8 neurons from 7 mice for each group). (F) AP threshold (*p* = 0.1094, Wilcoxon test). (G) AP amplitude (*p* = 0.1976, paired *t*‐test). (H) AP half‐width (*p* = 0.8497, paired *t*‐test). (I) Representative traces showing the effect of ZD7288 (10 μM) on spike of LHb neurons responded to 40 pA, 60 pA and 80 pA. (J) Frequency‐current (F–I) curves showed effect of ZD7288 (10 μM) on number spikes of LHb neurons respond to a series of 1 s current pulses from 0 pA to 100 pA with 20 pA steps (*n* = 8 neurons from 7 mice for each group, *F*_(5,35)_ = 2.412, *p*_(40 pA)_ = 0.0141, *p*_(60 pA)_ = 0.0007, *p*_(80 pA)_ < 0.0001, two‐way ANOVA). Data are shown as mean ± SEM. *p < 0.05, **p < 0.01，***p < 0.001.

Subsequently, we employed electrophysiological recordings to specifically investigate how the abundantly expressed HCN channels modulate the LHb neuronal excitability. Based on the absence or presence of Ih, we categorized the LHb neurons into two groups—namely, those without Ih and those with Ih (Figure 5A), and separately analyzed the proportion of three different firing types of neurons without Ih and with Ih in the LHb. Compared with neurons with Ih, the percentage of burst‐firing neurons and tonic‐firing neurons were higher, but silent‐firing neurons were lower in those LHb neurons without Ih (Figure 5B). Furthermore, our findings indicated that in tonic‐firing mode, the LHb neurons with Ih displayed a higher spontaneous firing rate than those without Ih (Figure 5C). Notably, although no substantial differences were observed in the RMP (Figure 5D) and rheobase current (Figure 5G) between these two groups, the LHb neurons with Ih exhibited a larger Cm and smaller Rin compared to neurons without Ih (Figure 5E,F). Moreover, five parameters related to active membrane properties, including the AP threshold, AP amplitude, RMP‐AP threshold, AP half‐width, and AHP amplitude (Figure 5H–L) were comparable between these two groups, while the LHb neurons with Ih generated more spikes in response to depolarizing‐current injections than those without Ih (Figure 5M,N). To further confirm the role of HCN channels in modulating the LHb neuronal excitability, we administered ZD7288, a selective HCN channel blocker, at a concentration of 10 μM during whole‐cell recordings (Figure 6A). Remarkably, the blockade of Ih by ZD7288 significantly decreased the spontaneous firing rate of the LHb tonic‐firing neurons (Figure 6B,C). Additionally, Ih blockade resulted in a more hyperpolarized RMP (Figure 6D) accompanied by a larger Rin (Figure 6E), but did not affect the AP threshold, AP amplitude, and AP half‐width (Figure 6F–H). Furthermore, the administration of ZD7288 significantly reduced the number of evoked action potential firing spikes in the LHb neurons (Figure 6I,J). In summary, these results provide a deeper understanding of the HCN channels' essential role in regulation of the LHb neuronal excitability.

### 3.4 Function of HCN channels and expression of HCN2 isoforms of the LHb up‐regulated in SNI 6w mice

**FIGURE 7:**
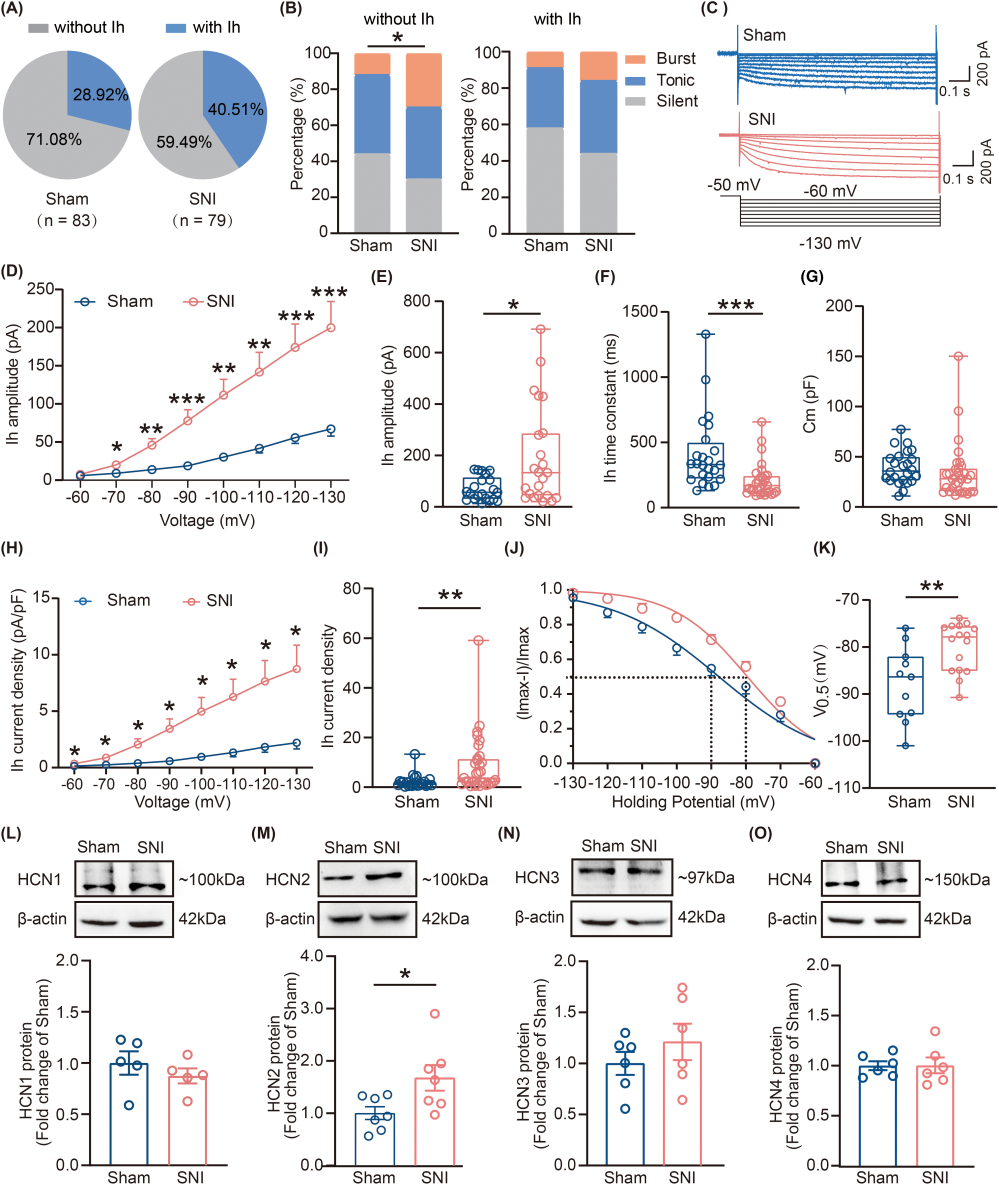
Increased Ih and up‐regulation of HCN2 isoforms of the LHb in SNI 6w mice. (A) Pie charts illustrating the percentage of LHb neurons without Ih and with Ih in Sham and SNI 6w mice (Sham, *n* = 59 neurons without Ih and 24 neurons with Ih from 36 mice; SNI, *n* = 47 neurons without Ih and 32 neurons from 17 mice, *p* = 0.1210, Chi‐square test). (B) Proportion of three firing types of neurons without Ih and neurons with Ih in the LHb of Sham 6w and SNI 6w mice, respectively (without Ih neurons in Sham 6w mice: *n* = 26 silent neurons, *n* = 26 tonic neurons, *n* = 7 burst neurons; without Ih neurons in SNI 6w mice: *n* = 14 silent neurons, *n* = 19 tonic neurons, *n* = 14 burst neurons from 29 mice, *p* = 0.0051, Chi‐square test; with Ih neurons in Sham 6w mice: *n* = 14 silent neurons, *n* = 8 tonic neurons, *n* = 2 burst neurons; with Ih neurons in SNI 6w mice: *n* = 14 silent neurons, *n* = 13 tonic neurons, *n* = 5 burst neurons from 21 mice, *p* = 0.1027, Chi‐square test.). (C) Representative traces of Ih currents of LHb neurons in Sham and SNI 6w mice. (D) The I‐V curves showing the amplitude of Ih responding to different voltages of LHb neurons in Sham and SNI 6w mice (Sham, *n* = 24 neurons from 12 mice; SNI, *n* = 31 neurons from 15 mice, *F*_(1.046,21.21)_ = 13.25, *p*_(−70 mV)_ = 0.0188, *p*_(−80 mV)_ = 0.0034, *p*_(−90 mV)_ = 0.0009, *p*_(−100 mV)_ = 0.0013, *p*_(−110 mV)_ = 0.0011, *p*_(−120 mV)_ = 0.0009, *p*_(−130 mV)_ = 0.0009, two‐way ANOVA). (E) Amplitude of Ih at −130 mV of LHb neurons in Sham and SNI 6w mice (Sham, *n* = 24 neurons from 12 mice; SNI, *n* = 31 neurons from 15 mice, *p* = 0.0049, Mann–Whitney test). (F) Time constant of Ih of LHb neurons in Sham and SNI 6w mice (Sham, *n* = 24 neurons from 12 mice; SNI, *n* = 31 neurons from 15 mice, *p* < 0.0001, Mann–Whitney test). (G) Cm of LHb neurons with Ih in Sham and SNI 6w mice (Sham, *n* = 24 neurons from 12 mice; SNI, *n* = 31 neurons from 15 mice, *p* = 0.0576, Mann–Whitney test). (H) Ih current density versus voltage in Sham and SNI 6w mice (Sham, *n* = 24 neurons from 12 mice; SNI, n = 31 neurons from 15 mice, *F*_(0.9957,20.20)_ = 7.095, *p*_(−60 mV)_ = 0.0282, *p*_(−70 mV)_ = 0.0252, *p*_(−80 mV)_ = 0.0140, *p*_(−90 mV)_ = 0.0106, *p*_(−100 mV)_ = 0.0142, *p*_(−110 mV)_ = 0.0147, *p*_(−120 mV)_ = 0.0133, *p*_(−130 mV)_ = 0.0141, two‐way ANOVA). (I) Ih current density at −130 mV in Sham and SNI 6w mice (Sham, *n* = 24 neurons from 12 mice; SNI, *n* = 31 neurons from 15 mice, *p* = 0.0019, Mann–Whitney test). (J) Activation curves in Sham and SNI 6w mice. (K) Half‐activation voltage of Ih in Sham and SNI 6w mice (Sham, *n* = 11 neurons from 8 mice; SNI, *n* = 16 neurons from 8 mice, *p* = 0.0079, Mann–Whitney test). (L–O), Representative images (UP) and quantitative analysis (Bottom) of western blot showing the protein level of HCN1‐4 from Sham and SNI mice. (L) HCN1 (*n* = 5 for each group, *p* = 0.3807, unpaired *t*‐test). (M) HCN2 (*n* = 7 for each group, *p* = 0.0305, unpaired *t*‐test). (N) HCN3 (*n* = 6 for each group, *p* = 0.3416, unpaired *t*‐test). (O) HCN4 (*n* = 6 for each group, *p* = 0.9736, unpaired *t*‐test). Data are shown as mean ± SEM or Min to Max. *p < 0.05, **p < 0.01, ***p < 0.001.

Considering our finding that HCN channels were crucial regulators of the LHb neuronal activity, we investigated whether increased LHb neuronal excitability in SNI 6w mice is associated with the HCN channelopathy. First, we performed in vitro electrophysiological recordings; intriguingly, results showed a notable rise in the proportion of Ih‐expressing neurons (Sham 6w, 28.92% vs SNI 6w, 40.51%) (Figure 7A). We further analyzed the correlation between the Ih and the LHb neuronal firing patterns. Results indicated that in LHb neurons without Ih, the proportion of bursting‐firing neurons significantly increased and that of silent‐firing neurons significantly decreased in SNI group. In LHb neurons with Ih, the proportion of burst‐firing neurons increased and that of silent‐firing neurons decreased in SNI 6w mice; however, this trend did not reach statistical significance (Figure 7B). Moreover, the amplitude of Ih recorded in the LHb neurons was higher in SNI 6w mice than in the Sham controls (Figure 7C–E). Additionally, compared with the Sham group, the activation of Ih was significantly accelerated in SNI group (Figure 7F). No difference was observed in Cm between the two groups (Figure 7G). Further analysis indicated that Ih current densities of the LHb neurons were greater in SNI 6w mice than in Sham mice (Figure 7H,I). By plotting normalized tail currents against voltage steps and fitting them with a Boltzmann function, we observed a depolarizing shift in the half‐activation voltage for Ih (V_0.5_) of the LHb neurons in SNI 6w mice (Figure 7J,K). Subsequently, we explored the expression of four HCN channel subunits in the LHb at the protein level. Western blot analysis revealed a 1.68 ± 0.25‐fold increase of HCN2 isoforms of the LHb in SNI group compared to Sham group. However, no significant differences were observed in the protein expression levels of HCN1, HCN3, and HCN4 isoforms of the LHb between these two groups (Figure 7L–O). Taken together, these findings indicate that enhanced function of HCN channels and increased expression of HCN2 isoforms of the LHb might potentially contribute to increased LHb neuronal excitability in SNI 6w mice.

### 3.5 Microinjection of ZD7288 into the LHb ameliorated pain and related depressive‐like behaviors in SNI 6w mice

**FIGURE 8:**
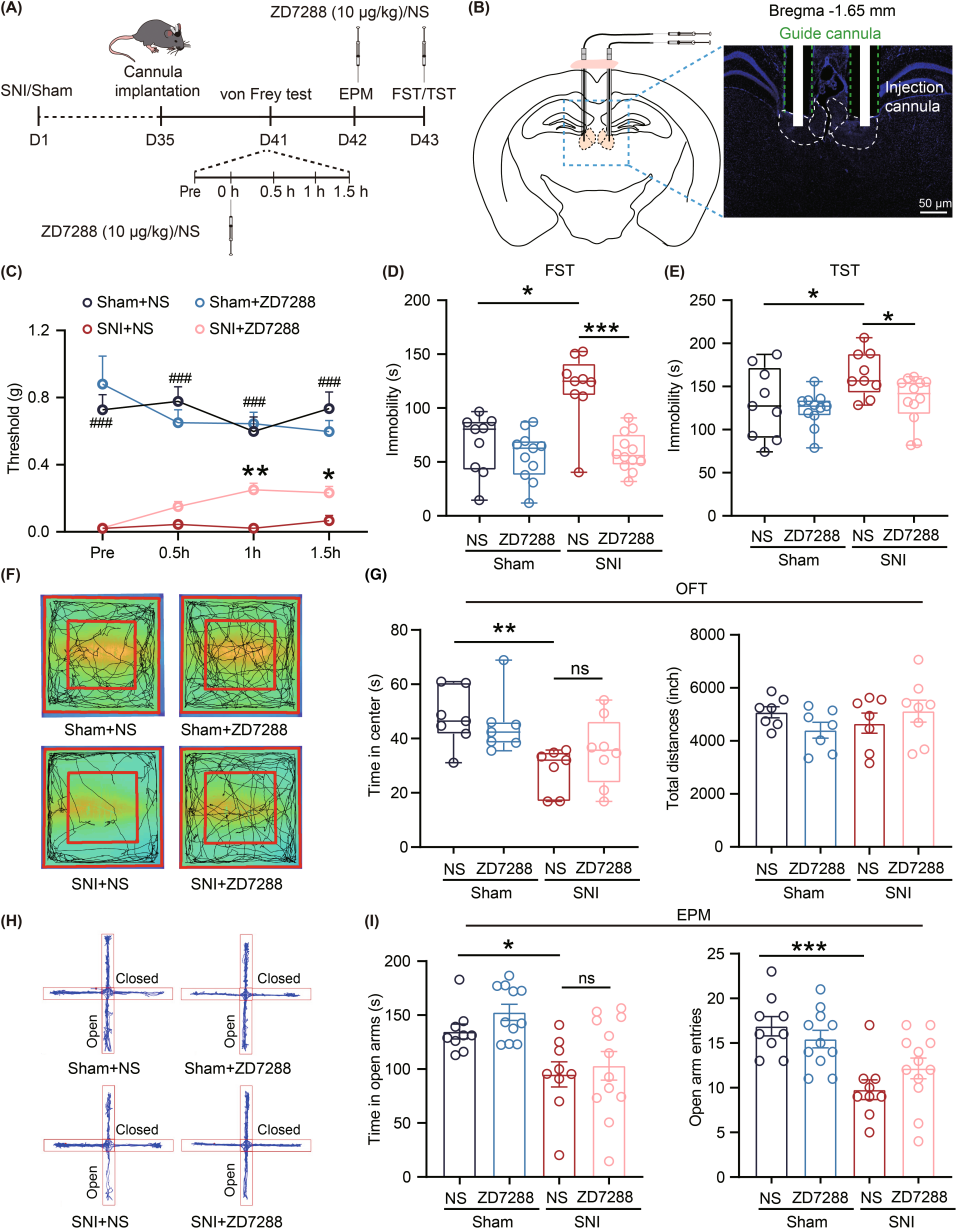
Bilateral microinjection of ZD7288 into the LHb attenuates pain and depressive‐like behaviors in SNI 6w mice. (A) Timeline of experimental procedures. (B) Schematic illustration for drug delivery. (C) Effects of local injection of ZD7288 (10 μg/kg) into LHb on mechanical pain threshold in Sham and SNI mice (Sham + NS, *n* = 9 mice; Sham + ZD7288, *n* = 11 mice; SNI + NS, *n* = 9 mice; SNI + ZD7288, *n* = 12 mice, *F*_(2.153,15.31)_ = 4.125, Sham + NS vs SNI + NS: ###*p* = 0.0003, *p*_(pre)_, *p*_(0.5h)_ < 0.0001, *p*_(1h)_ = 0.0008, *p*_(1.5h)_ = 0.0002; SNI + NS vs SNI + ZD7288: *p < 0.05, **p < 0.01, *p*_(pre)_ = 0.4777, *p*_(0.5h)_ = 0.0167, *p*_(1h)_ = 0.0013, *p*_(1.5h)_ = 0.0099, Two‐way ANOVA). (D, E) Effects of local injection of ZD7288 (10 μg/kg) into LHb on immobility time in FST and TST (Sham + NS, *n* = 9 mice; Sham + ZD7288, *n* = 11 mice; SNI + NS, *n* = 9 mice; SNI + ZD7288, *n* = 12 mice). (D) FST (Sham + NS vs SNI + NS: *p* = 0.0173; Sham + ZD7288 vs SNI + NS: *p* = 0.0006; SNI + NS vs SNI + ZD7288: *p* = 0.008, Kruskal–Wallis test). (E) TST (Sham + NS vs SNI + NS: *p* = 0.0336; Sham + ZD7288 vs SNI + NS: *p* = 0.0021; SNI + NS vs SNI + ZD7288: *p* = 0.0498, Kruskal–Wallis test). (F) Representative images of activity tracking of mice in OFT (Sham + NS, *n* = 7 mice; Sham + ZD7288, *n* = 7 mice; SNI + NS, *n* = 7 mice; SNI + ZD7288, *n* = 8 mice). (G) Left, time in center region within 5 min in OFT (Sham + NS vs SNI + NS, *p* = 0.0023; Sham + ZD7288 vs SNI + NS, *p* = 0.0063; SNI + NS vs SNI + ZD7288, *p* = 0.1733, Kruskal–Wallis test). Right, total distance traveled within 30 min in OFT (*F*_(3,25)_ = 1.016, Sham + NS vs SNI + NS, *p* = 0.4123, Sham +ZD7288 vs SNI + NS, *p* = 0.5923, SNI + NS vs SNI + ZD7288, *p* = 0.3499, one‐way ANOVA). (H) Representative activity tracking of mice in EPM within 5 min (Sham + NS, *n* = 9; Sham + ZD7288, *n* = 11; SNI + NS, *n* = 9; SNI + ZD7288, *n* = 12). (I) Left, time in open arms (*F*_(3,29.90)_ = 7.029, Sham + NS vs SNI + NS, *p* = 0.0115; Sham + ZD7288 vs SNI + NS, *p* = 0.0010; SNI + NS vs SNI + ZD7288, *p* = 0.6563, Brown‐Forsythe ANOVA test). Right, Open arms entries (*F*_(3,37)_ = 7.898, Sham + NS vs SNI + NS, *p* = 0.0001; Sham +ZD7288 vs SNI + NS, *p* = 0.0009; SNI + NS vs SNI + ZD7288: *p* = 0.1308, one‐way ANOVA). Data are shown as mean ± SEM or Min to Max. *p < 0.05, **p < 0.01, ***p < 0.001, ###*p* < 0.001.

To determine whether the HCN channelopathy in the LHb is responsible for CADS in chronic neuropathic pain, a double‐guided cannula was bilaterally implanted into the LHb at 5w after the SNI surgery (Figure 8A). One week later, pain and anxiodepressive‐like behaviors were evaluated followed by the local infusion of either NS or ZD7288 (10 μg/kg, 200 nL on each side) into the LHb of SNI and Sham mice (Figure 8B).^39^ Results showed that compared with the NS‐infused group, the mechanical pain thresholds were significantly increased after the infusion of ZD7288 into the LHb at both 60 and 90 min in SNI 6w mice (Figure 8C). Furthermore, the application of ZD7288 produced evident antidepressant effects in both FST and TST (Figure 8D,E) and did not affect the motor activity of mice, as evaluated by the OFT (Figure 8F,G). Notably, no significant differences were observed in the time spent in the center zone (as assessed by the OFT), or in the time spent in open arms and open‐arm entries (as assessed by EPM) between the ZD7288‐infused and the NS‐infused mice (Figure 8H,I). Thus, we propose that enhanced function of HCN channels in the LHb plays a critical role in the development of chronic neuropathic pain and comorbid depressive‐like behaviors.

### 3.6 Specific HCN2 channels knockdown decreased the LHb neuronal excitability and improved pain and related depressive‐like behaviors in SNI 6w mice

**FIGURE 9:**
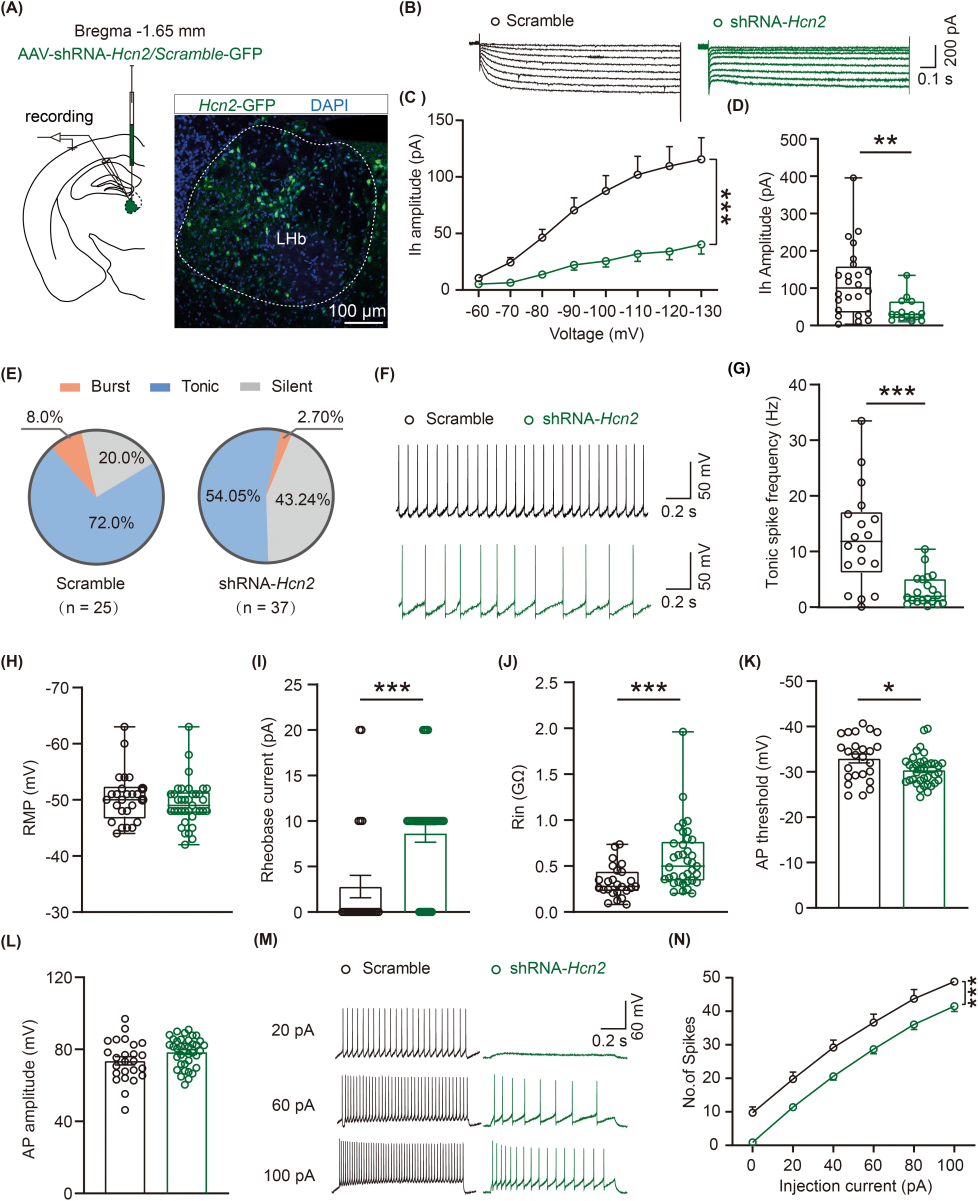
Specific knockdown of HCN2 channels decreases the LHb neuronal excitability. (A) Schematic illustration for virus injection (left), representative figure for virus injection in the LHb (right) (scale bar, 100 μm). (B) Representative traces of Ih in the LHb neurons. (C) The I‐–V curves showing the amplitude of Ih responding to different voltages of LHb neurons in virus injection mice (scramble, *n* = 24 neurons from 8 mice; SNI, *n* = 13–15 neurons from 8 mice, *F*_(7.256)_ = 8.625, *p*_(−60 mV)_ = 0.0309, *p*_(−70 mV)_ = 0.0004, *p*_(−80 mV)_ = 0.0002, *p*_(−90 mV)_ = 0.0003, *p*_(−100 mV)_ = 0.0002, *p*_(−110 mV)_ = 0.0004, *p*_(−120 mV)_ = 0.0003, *p*_(−130 mV)_ = 0.0011, two‐way ANOVA). (D**)** Amplitude of Ih at −130 mV of LHb neurons in scramble and shRNA‐*Hcn2* mice (scramble, *n* = 24 neurons from 8 mice; SNI, *n* = 15 neurons from 8 mice, *p* = 0.0036, Mann–Whitney test). (E) Pie charts showing the percentage of three firing types of LHb neurons in scramble and shRNA‐*Hcn2* mice (scramble, *n* = 2 burst neurons, 18 tonic neurons and 5 silent neurons from 8 mice; shRNA‐*Hcn2*, *n* = 1 burst neurons, 20 tonic neurons, and 16 silent neurons from 8 mice, *p* = 0.1334, Chi‐square test). (F, G) Representative traces (F) and statistical data (G) of spontaneous firing frequency of the LHb tonic‐firing neurons in scramble and shRNA‐*Hcn2* mice (scramble, *n* = 18 neurons from 8 mice; SNI, *n* = 20 neurons from 8 mice, *p* = 0.0002, Mann–Whitney test). H–J, Summary of the passive membrane properties of LHb neurons in scramble and shRNA‐*Hcn2* mice (scramble, *n* = 25 neurons from 8 mice, shRNA‐*Hcn2*, *n* = 37 neurons from 8 mice). (H) RMP (*p* = 0.3442, Mann–Whitney test). (I) Rheobase current (*p* = 0.0001, Mann–Whitney test). (J) Rin (*p* = 0.0002, Mann–Whitney test). (K, L) Summary of the active membrane properties of LHb neurons in scramble and shRNA‐*Hcn2* mice (scramble, *n* = 25 neurons from 8 mice, shRNA‐*Hcn2*, n = 37 neurons from 8 mice). (K) AP threshold (*p* = 0.0183, unpaired *t*‐test). (L) AP amplitude (*p* = 0.0556, unpaired *t*‐test). (M, N) Representative traces of the spike of LHb neurons responded to 20 pA, 60 pA and 100 pA in scramble and shRNA‐*Hcn2* mice. (M) Frequency‐current (F–I) curves showing the number of spikes of LHb neurons responded to a series of 1 s current pulses from 0 pA to 100 pA with 20 pA steps in scramble and shRNA‐*Hcn2* mice (scramble, *n* = 25 neurons from 8 mice, shRNA‐*Hcn2*, *n* = 37 neurons from 8 mice, *F*_(5,300)_ = 0.9407, *p*_(0 pA)_ < 0.0001, *p*_(20 pA)_ = 0.0008, *p*_(40 pA)_ = 0.0012, *p*_(60 pA)_ = 0.0081, *p*_(80 pA)_ = 0.0201, *p*_(100 pA)_ = 0.0405, two‐way ANOVA). Data are shown as mean ± SEM or Min to Max. *p < 0.05, **p < 0.01, ***p < 0.001.

Although our findings revealed that inhibition of the LHb HCN channels can exert evident inhibitory effects on the LHb neuronal excitability and further produce analgesic and antidepressant effects in SNI 6w mice, the specific role of the HCN2 channel has not yet been clarified. To address this issue, we selectively downregulated the HCN2 channels' expression in the LHb neurons by virus injection (Figure 9A) and explored how reduced expression of HCN2 channels regulate the LHb neuronal excitability. We employed in vitro electrophysiological recordings on GFP‐positive neurons distinguished by 470‐nm LED light. Electrophysical results showed that shRNA‐*Hcn2* greatly decreased the Ih amplitude of LHb neurons (Figure 9B). Furthermore, we observed HCN2 channels knockdown evidently decreased the proportion of burst‐firing neurons (shRNA‐Scramble, 8.0%; shRNA‐*Hcn2*,2.70%) and tonic‐firing neurons (shRNA‐Scramble, 72.00%; shRNA‐*Hcn2*,54.05%) and increased the proportion of silent‐firing neurons (shRNA‐Scramble, 20.00%; shRNA‐*Hcn2*,43.34%) in the LHb (Figure 9E). Besides, specific HCN2 channels knockdown significantly decreased spontaneous firing frequency of tonic‐firing neurons in the LHb (Figure 9F,G). Although the down‐regulation of HCN2 channels had no effects on neuronal RMP (Figure 9H) and AP amplitude (Figure 9L) of the LHb neurons, specific HCN2 channels knockdown significantly increased the rheobase current (Figure 9I) and Rin (Figure 9J) and obviously depolarized the neuronal AP threshold of the LHb neurons. Notably, blocking HCN2 channels by genetic knockdown remarkably decreased the number of evoked AP firing spikes (Figure 9M,N).

**FIGURE 10:**
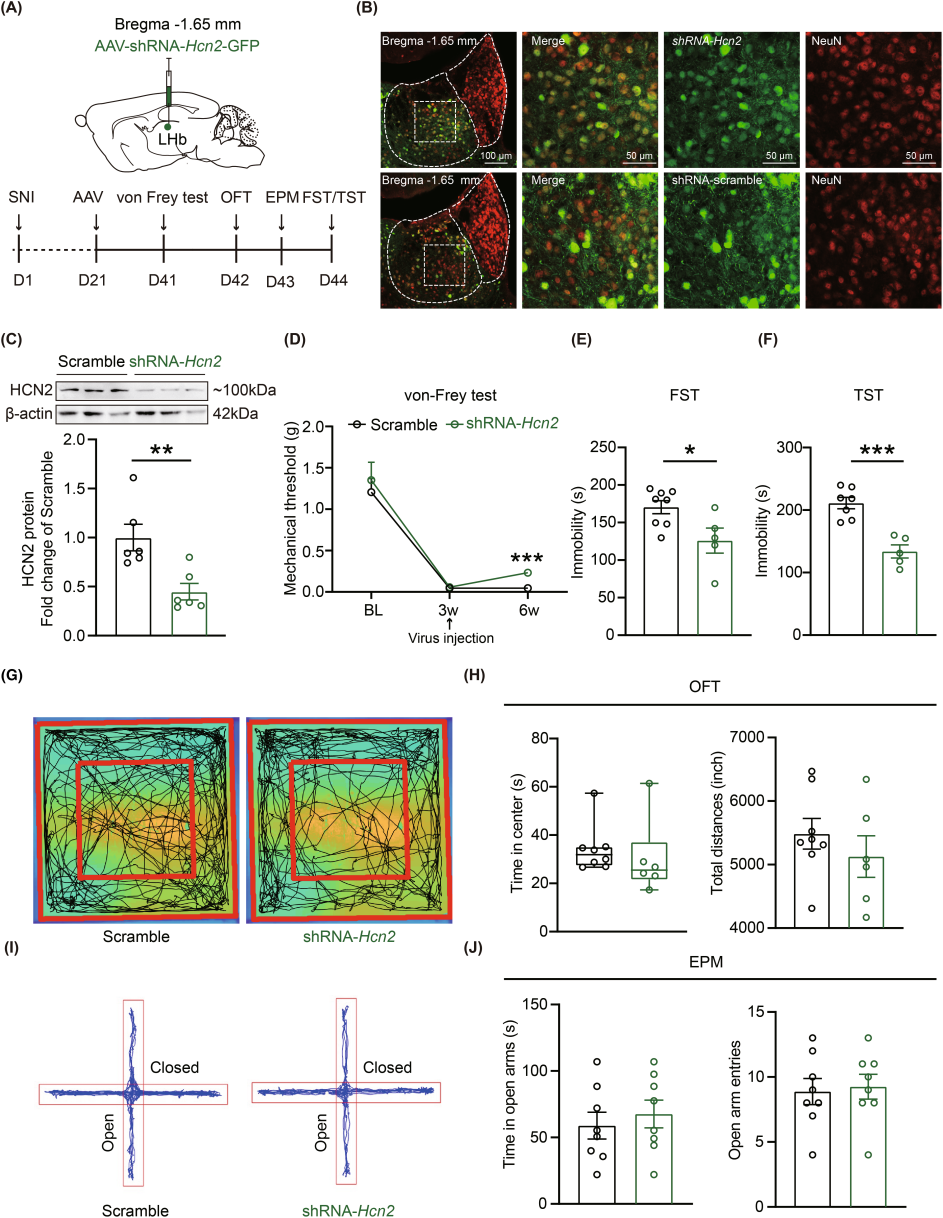
Bilateral knockdown of HCN2 channels in the LHb ameliorate pain and comorbid depressive‐like behaviors in SNI 6w mice. (A) Schematic illustration for virus injection and timeline of experimental procedures. (B) Representative and magnified images of AAV‐shRNA‐*Hcn2*‐GFP or AAV‐shRNA‐scramble‐GFP co‐labeled with NeuN in the LHb. (C) Representative images (UP) and quantitative analysis (Bottom) of Western blot showing the protein level of HCN2 from the LHb of scramble and shRNA‐*Hcn2* mice (*n* = 6 for each group, *p* = 0.0061, unpaired *t* test). (D) Effects of the HCN2 knockdown on mechanical pain threshold in scramble and shRNA‐*Hcn2* mice (scramble, *n* = 16 mice, shRNA‐*Hcn2*, *n* = 11 mice, *F*_(2,59)_ = 0.1924, *p*_(6w)_ = 0.0006, Two‐way ANOVA). (E, F) Effects of the HCN2 knockdown on immobility time in FST and TST. (E) FST (scramble, *n* = 8 mice, shRNA‐*Hcn2*, *n* = 5 mice, *p* = 0.0236, unpaired *t* test). (F) TST (scramble, *n* = 7 mice, shRNA‐*Hcn2*, *n* = 5 mice, *p* = 0.0002, unpaired *t* test). (G) Representative images of activity tracking of mice in OFT. (H) Left, time in center region with 5 min in OFT (scramble, *n* = 8 mice, shRNA‐*Hcn2*, *n* = 6 mice, *p* = 0.1419, Mann–Whitney test). Right, total distance traveled within 30 min in OFT (scramble, *n* = 8 mice, shRNA‐*Hcn2*, *n* = 6 mice, *p* = 0.3831, unpaired *t* test). (I) Representative activity tracking of mice in EPM within 5 min. (J) Left: time in open arms (scramble, *n* = 8 mice, shRNA‐*Hcn2*, *n* = 8 mice, *p* = 0.5555, unpaired *t* test). Right: open arms entries (scramble, *n* = 8 mice, shRNA‐*Hcn2*, *n* = 8 mice, *p* = 0.7914, unpaired *t* test). Data are shown as mean ± SEM or Min to Max. *p < 0.05, **p < 0.01, ***p < 0.001.

Based on these observations from electrophysiological experiments, we further explored the effects of the LHb specific HCN2 channels knockdown on the mice's behaviors. AAV‐shRNA‐*Hcn2*‐GFP or AAV‐shRNA‐scramble‐GFP was bilaterally injected into the LHb. After 3 weeks for full virus expression (Figure 10A,B), the protein levels of HCN2 channels were significantly decreased (Figure 10C). Various behavioral tests were performed to evaluate mechanical pain threshold and anxiodepressive‐like behaviors in SNI 6w mice. Noteworthily, after 3 weeks of virus injection, the specific knockdown of HCN2 channels in the LHb obviously improved SNI‐induced mechanical allodynia (Figure 10D) and further reduced depressive‐like behaviors in SNI 6w mice, which exhibited decreased immobility time in FST (Figure 10E) and TST (Figure 10C). However, this HCN2 channels' downregulation in the LHb exerted no effects on anxiety‐like behavior in SNI 6w mice in OFT (Figure 10G,H) or EPM (Figure 10I,J). In summary, our results revealed that the HCN2 channels in the LHb were key factors underlying chronic pain and associated depressive‐like behaviors.

## 4 DISCUSSION

In this study, we identified a significant relationship between the HCN channelopathy, especially the HCN2 channelopathy, and the hyperactivation of LHb neurons. This relationship is crucial for understanding the development of chronic neuropathic pain and comorbid depressive‐like behaviors. We found that all four HCN channel subtypes were expressed in the LHb neurons at the protein level and exhibited distinct expression patterns. Notably, Ih‐expressing neurons showed higher intrinsic neuronal excitability than neurons lacking Ih. Furthermore, we observed enhanced function of HCN channels and increased expression of HCN2 isoforms in the LHb of SNI 6w mice. Either pharmacological blockade or virus‐specific knockdown of HCN2 channels in the LHb was sufficient to decrease the LHb neuronal excitability and produced analgesic and antidepressant effects.

Several studies have reported that different chronic pain models can induce adverse psychological consequences, such as major depression and anxiety at varying time points. For instance, animals with chronic neuropathic pain induced by partial infraorbital nerve transection^7^ or chronic constriction injury (CCI) of the sciatic nerve^26^ showed anxiodepressive‐like behaviors at 4w after surgery. While mice with SNI‐induced neuropathic pain exhibited anxiodepressive‐like behaviors at 6w after the SNI surgery.^12^, ^27^ Moreover, animals with chronic inflammatory pain induced by complete Freund's adjuvant (CFA) display anxiodepressive‐like behaviors at 3w after the CFA injection.^10^, ^27^ Consistent with previous studies, we also observed evident anxiodepressive‐like behaviors in SNI 6w mice. Analytically, the time point at which negative emotions occur in chronic pain may depend on the causes and intensity of pain and be regulated by different neural mechanisms. Noteworthily, various studies classified SNI animals into pain with or without anhedonia (a depressive phenotype) according to the mice performance in SPT, and implied this individual difference in pain‐induced anhedonia was closely related to brain‐derived neurotrophic factor (BDNF)–tropomyosin‐receptor‐kinase B (TrkB) signaling in the medial prefrontal cortex (mPFC).^44^, ^45^ Recently, this BDNF–TrkB signaling in thalamic was verified to play a key role in the mechanism of resilience against stress.^46^ Whether the LHb underlines the psychological resilience to anxiodepressive‐like behaviors in mice with chronic pain needs to be further investigated.

Evidence from both human studies and preclinical animal research suggests that increased neuronal activity in the LHb neurons plays a critical role in depression and chronic pain.^21^, ^26^, ^47^ Our in vitro electrophysiological recordings further supported this notion, as we observed an increased percentage of the LHb burst‐firing neurons and a higher spike frequency in both spontaneous and evoked firing activity of the LHb neurons in SNI 6w mice, which is consistent with a previous study that also illustrated an increase in evoked AP spikes in the LHb neurons of SNI 6w mice.^27^ Additionally, we observed a hyperpolarized RMP in all recorded LHb neurons as well as silent neurons in SNI 6w mice. Since the LHb burst‐firing has been demonstrated to depend on a more hyperpolarized RMP,^18^ and our results also indicated that the RMP of the LHb burst‐firing neurons was more hyperpolarized than that of silent and tonic‐firing neurons. Thus, we speculated that increased percentage of burst‐firing neurons and hyperpolarization of silent‐firing neurons in SNI 6w mice can be attributed primarily to the hyperpolarization of RMP.

As previously indicated, the neuronal activity is influenced by various basic membrane properties. Except for RMP, Rin is correlated with the regulation of neuronal activity and is closely associated with HCN channels. However, the functional modulation of Rin by HCN channels and its subsequent impact on neuronal excitability remains controversial. Several studies have demonstrated that dysfunction of HCN channels increases membrane Rin and neuronal excitability. For instance, knockout of HCN1 in dorsal CA1 pyramidal neurons induced an increase in Rin, along with higher frequency spike activity.^48^ Similarly, Yi et al.^49^ reported that reduced function of HCN2 causes a larger Rin and hyperexcitability in human H1 embryonic stem cells and hippocampal neurons. By contrast, Zhu et al.^43^ found that, although HCN2 knockout increased Rin in thalamocortical neurons from the ventral basal thalamus, HCN channels' impairment suppresses neuronal excitability. Here, our electrophysiological results implied that the LHb neurons with Ih exhibited a smaller Rin and higher spontaneous firing and evoked AP firing spikes. Both pharmacological inhibition of HCN channels by ZD7288 perfusion and specific knockdown of HCN2 channels could significantly increase neuronal Rin and decrease the excitability of LHb neurons, especially decreased spontaneous firing frequency of tonic‐firing neurons. Therefore, discrepancies observed between our observations and those of others suggested that HCN channels in different brain regions may affect the electrophysiological properties of neurons in an opposite way. Noteworthily, the LHb neurons without Ih showed higher percentage of burst‐firing neurons than those neurons with Ih, and while inhibition of Ih significantly suppressed the neuronal excitability of tonic‐firing neurons, the activity of burst‐firing neurons was mildly affected. Thus, combined with previous study^21^ and our observations, we speculated that burst‐firing neurons were primarily regulated by low‐voltage‐sensitive T‐type calcium channels (T‐VSCCs) but tonic‐firing neurons were predominantly regulated by HCN channels.

Numerous studies have indicated that alterations in the function and expression of HCN channels in different brain regions exert diverse effects on neuronal excitability and play a key role in various neurological disorders, particularly in pain,^50^, ^51^ epilepsy,^52^ cognitive impairment,^49^ and affective disorders such as anxiety^53^ and depression.^54^ Intriguingly, an increase in the expression of HCN2 isoforms and neuronal AP firing frequency has been observed in the periaqueductal gray matter^50^ and dorsal root ganglion (DRG) neurons^55^ following neuropathic or inflammatory pain. By contrast, a decrease in the expression of HCN2 isoforms and neuronal excitability has been noted in cholinergic interneurons of the nucleus accumbens shell^51^ and dopamine neurons of the ventral tegmental area (VTA)^53^ under depression. Thus, it is evident that HCN channels exert significant and diverse effects on the regulation of pain perception and affective behaviors across different brain regions. In this study, we revealed that the LHb neuronal excitability was increased in SNI 6w mice, and illustrated that the SNI‐induced hyperactivity of the LHb neurons was attributed to enhanced function of HCN channels and increased expression of HCN2 channels in the LHb. Both bilateral microinjection of ZD7288 into the LHb and specific knockdown of HCN2 channels significantly improved SNI‐induced mechanical allodynia and comorbid depressive‐like behaviors. Our findings establish a positive correlation between HCN channels, especially HCN2 channels, and increased LHb neuronal excitability which contributes to chronic pain and comorbid depressive‐like behaviors. Intriguingly, HCN channels' inhibition had no effects on SNI‐induced anxiety‐like behaviors. We speculated that HCN channels in the LHb serve a crucial role in regulating depressive‐like behaviors, while anxiety‐like behaviors in SNI 6w mice might be associated with other neural mechanisms in the LHb or related to other brain regions, such as the amygdala,^56^, ^57^, ^58^, ^59^ hippocampus,^60^ and prefrontal cortex.^61^, ^62^ Further investigations were required to confirm these speculations.

Previous studies have identified several molecules involved in regulating the activity of HCN channels, including tetratricopeptide‐repeat‐containing Rab8b‐interacting protein (TRIP8b)^63^ and cyclic adenosine monophosphate (cAMP).^64^ TRIP8b interacts with the HCN pore‐forming subunits at multiple interaction sites and differentially regulates the function and subcellular distribution of HCN channels.^65^ Reduced expression of TRIP8b or loss of TRIP8b phosphorylation can disrupt TRIP8b‐HCN interactions and lead to a reduction in the HCN current.^65^, ^66^, ^67^ Behaviorally, the restoration of TRIP8b in the hippocampus has been demonstrated to reverse the impaired HCN channel trafficking and hence produce antidepressant effects in TRIP8b KO mice. Additionally, cAMP is known to be a potent regulator of HCN channels’ function as it can enhance the channel open probability and increase the frequency of current at any given voltage. Pathophysiologically, cAMP‐mediated modulation of HCN channels activity contributes to the development of various neurological disorders. For example, the HCN2 activation resulting from cAMP elevation underlies DRG neuronal hyperexcitability in inflammatory and neuropathic pain,^55^ whereas the suppression of cAMP‐dependent HCN channel gating in the bed nucleus of the stria terminalis alleviates stress responses.^68^ Interestingly, recent studies have demonstrated contrasting modulatory effects of chronically altered cAMP on HCN channels activity in pyramidal neurons from dorsal hippocampal and dopamine neurons from VTA.^64^, ^69^ Therefore, investigating whether cAMP or TRIP8b participates in the HCN channelopathy of the LHb under the comorbidity of SNI‐induced chronic pain and major depression and elucidating their interactions with HCN channels are important areas for future research aimed at elucidating the central mechanisms underlying the pathogenesis of CADS in chronic pain.

Notably, the regulation of CADS in chronic pain does not only relies on a specific brain region but also is determined by intricate interactions of a complex neuronal network. Recent advancements in the study of neural circuits have significantly contributed to our understanding of anatomical and functional connections in the LHb. The LHb receives diverse inputs from a wide range of brain regions, including the hypothalamic structures,^38^ ventral pallidum,^22^ thalamus,^70^ and amygdala.^27^ Regarding efferent projections, the LHb plays a crucial role in transmitting signals to the dopaminergic and serotonergic system, such as the VTA and dorsal raphe nucleus.^23^, ^71^ These intricate neural circuits associated with the LHb have been extensively implicated in depressive‐like behaviors and are strongly correlated with the hyperexcitability of LHb neurons. To advance our understanding of the role of HCN channels, especially HCN2 channels, in the LHb in pain‐related anxiodepressive‐like manifestations, future studies were required to delineate how the hyperexcitability of LHb neurons under CADS in chronic pain reshapes the LHb output.

## 5 CONCLUSION

**FIGURE 11:**
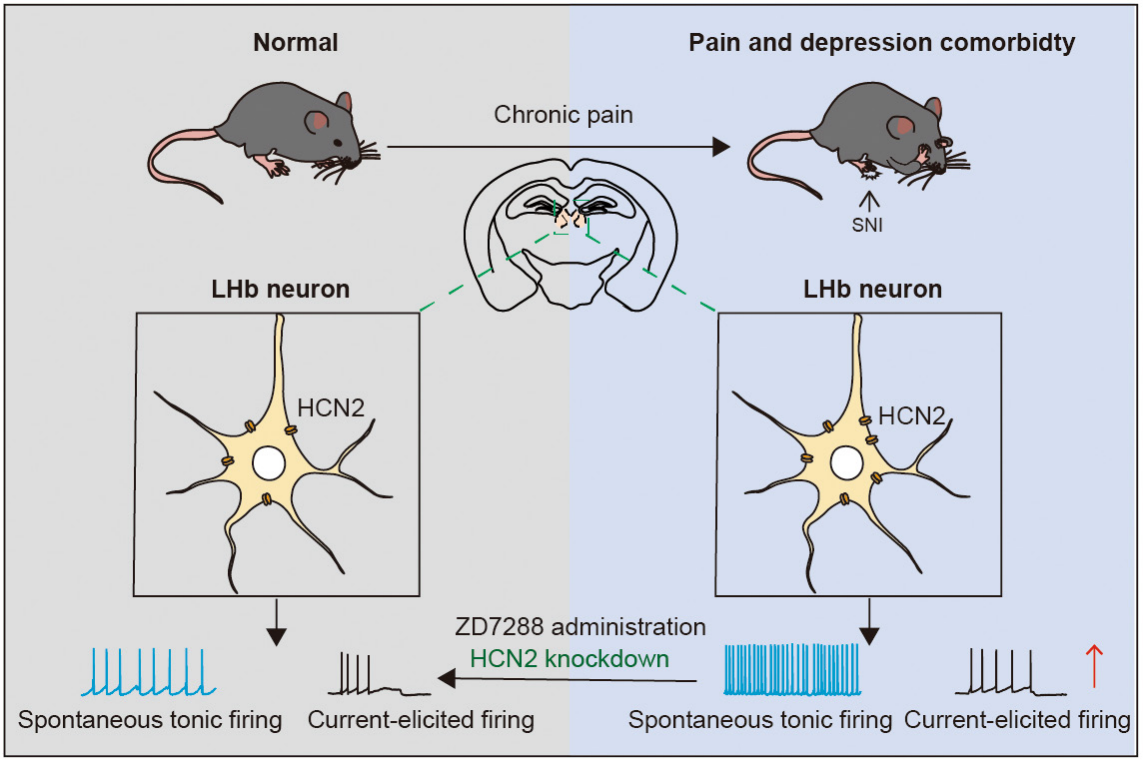
Graphical abstract of this study. LHb, Lateral habenula; HCN, Hyperpolarization‐activated cyclic nucleotide‐gated channels; SNI, Spared Nerve Injury.

In conclusion, our findings highlight that increased LHb neuronal excitability, which—at least partially—results from enhanced function of HCN channels and increased expression of HCN2 isoforms, is crucial for the development of CADS in chronic pain. The suppression of HCN channels in the LHb via local administration of ZD7288 and specific HCN2 channel knockdown evidently decreased the LHb neuronal excitability and produced significant analgesic and antidepressant effects in those CADS mice (Figure 11). These findings expanded our understanding of the cellular mechanisms underlying CADS in chronic pain and provided novel insights for developing optimal treatments for patients who suffered from this comorbidity.

## AUTHOR CONTRIBUTIONS

Zhang D, Liu T, and Zhang X interpreted the data and designed and supervised the study. Cao X performed all behavioral assays, virus injection, western blot, and data analysis. Zhu M, Yan Y, and Cao X performed the electrophysiological experiments and data analysis. Xu G and Li F performed the cannula infusion experiments. Zhang J and Wang J performed immunohistochemical experiments and data analysis. Cao X wrote the manuscript, with the collaboration of Zhu M, Wang J, Xu G, Li F, and Zhang J. Liu T revised the manuscript. All authors discussed the data and approved the final manuscript.

## FUNDING INFORMATION

This study was supported by the National Natural Science Foundation of China (81860216 to Zhang D, 32060186 to Liu T, 82260225 and 81903595 to Zhu M, and 81960216 to Zhang X), the Natural Science Foundation of Jiangxi Province (20202BAB216043 to Zhu M), and Gan Po Talent Support Program—Academic and technical leaders training program in major disciplines of Jiangxi Province (20232BCJ23040 to Zhu M) and Jiangxi Province Postgraduate Innovation Special Fund Project (YC2023‐B057 to Cao X).

## CONFLICT OF INTEREST STATEMENT

All authors of this study declared that there were no conflicts of interest.

## Supporting information


Appendix S1.

## Data Availability

The data that support the findings of this study are available from the corresponding author upon reasonable request.
