# Supplementary material for: HCN channels in the lateral habenula regulate pain and comorbid depressive‐like behaviors in mice
Source: CNS Neurosci Ther. 2024 Jul 3;30(7):e14831. doi: 10.1111/cns.14831 (PMC11222070; doi:10.1111/cns.14831)
Supplement: Supplementary file 1 — Appendix S1. [file CNS-30-e14831-s001.zip › cns14831-sup-0001-Supplemental_Files_1.pdf]

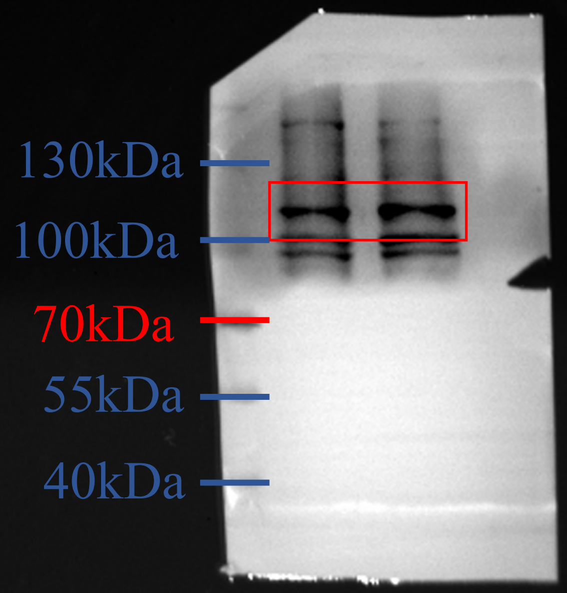

Full unedited blot for Figure7A (HCN1)

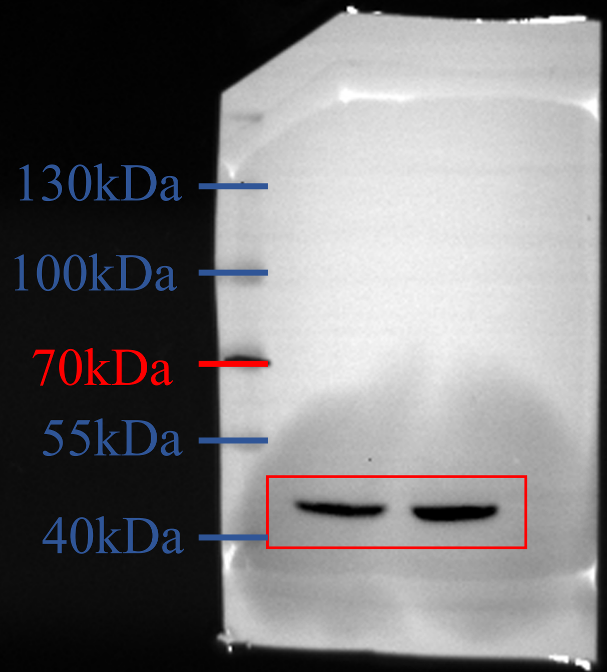

Full unedited blot for Figure7A (β-actin)

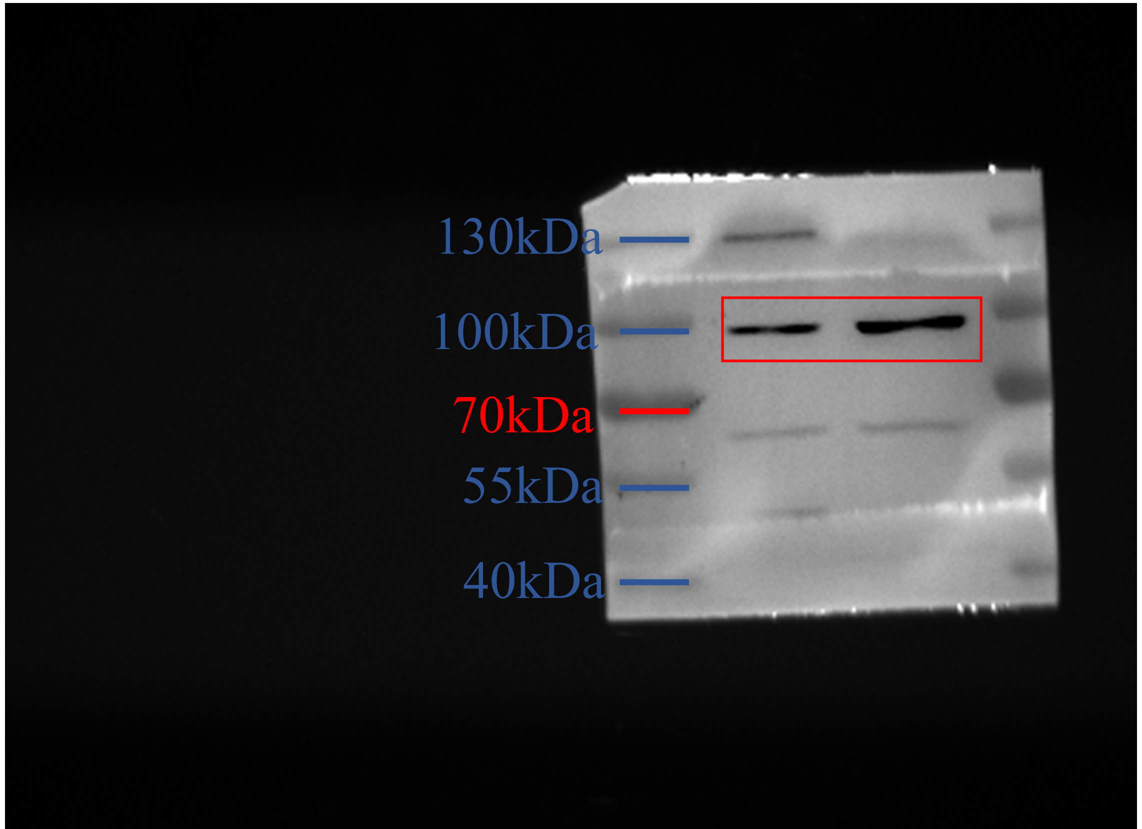

Full unedited blot for Figure7B (HCN2)

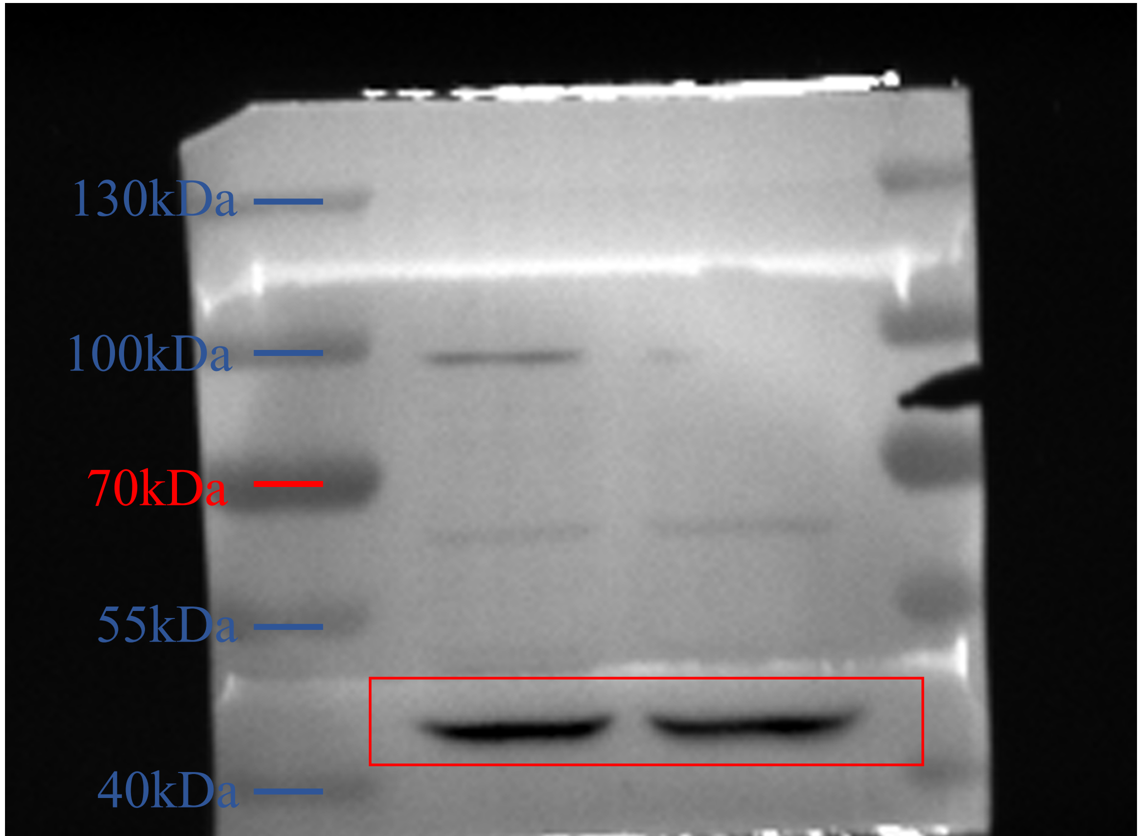

Full unedited blot for Figure7B (β-actin)

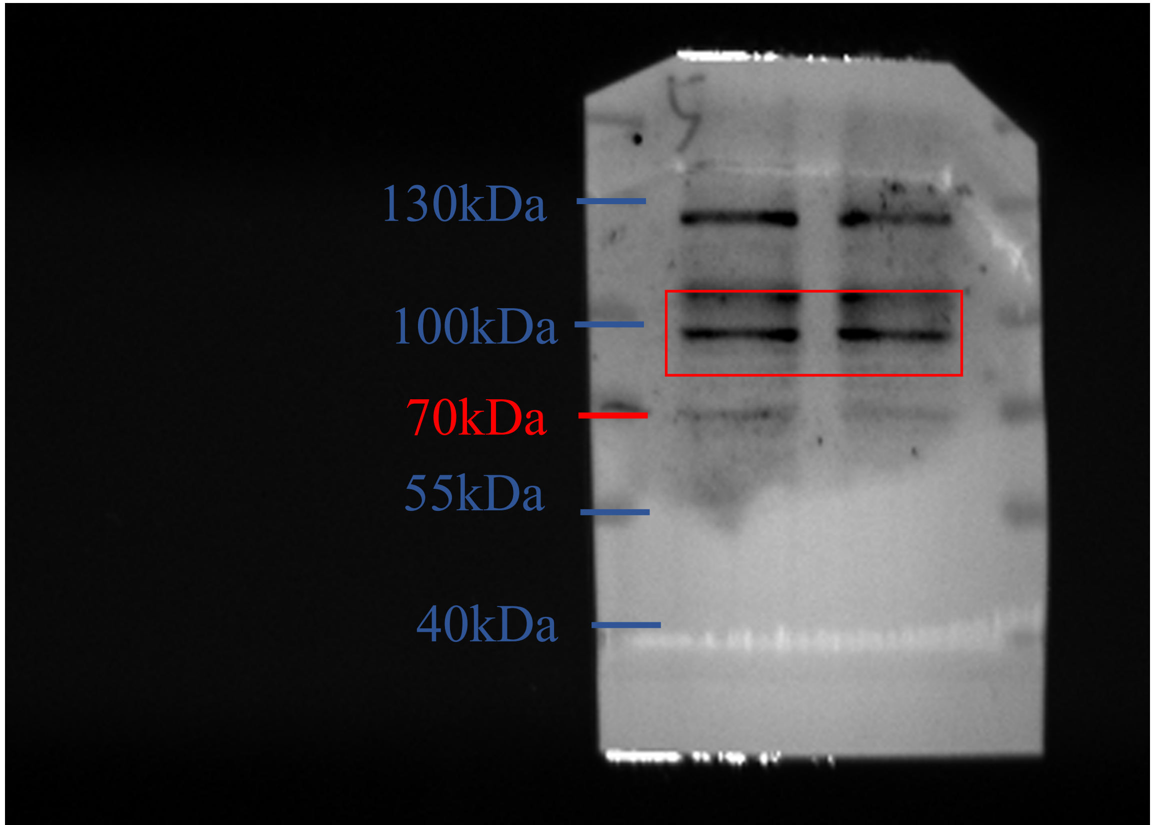

Full unedited blot for Figure7C (HCN3)

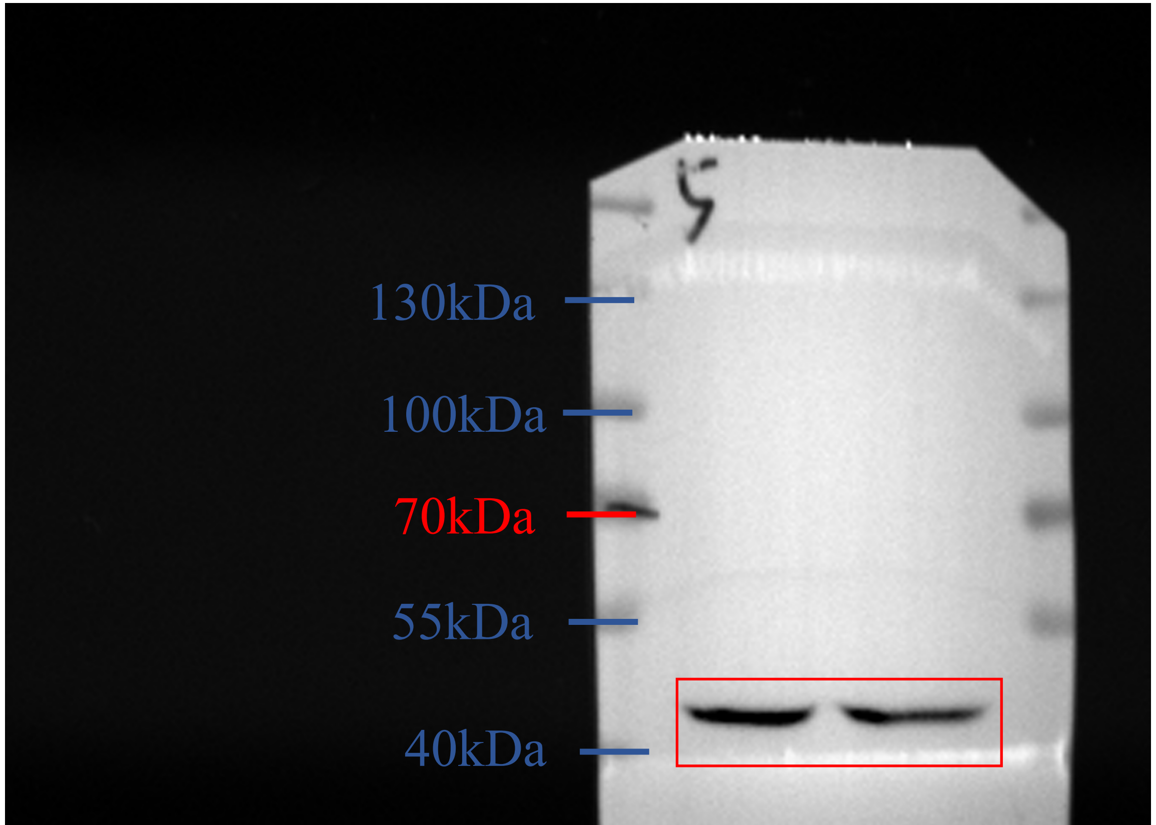

Full unedited blot for Figure7C (β-actin)

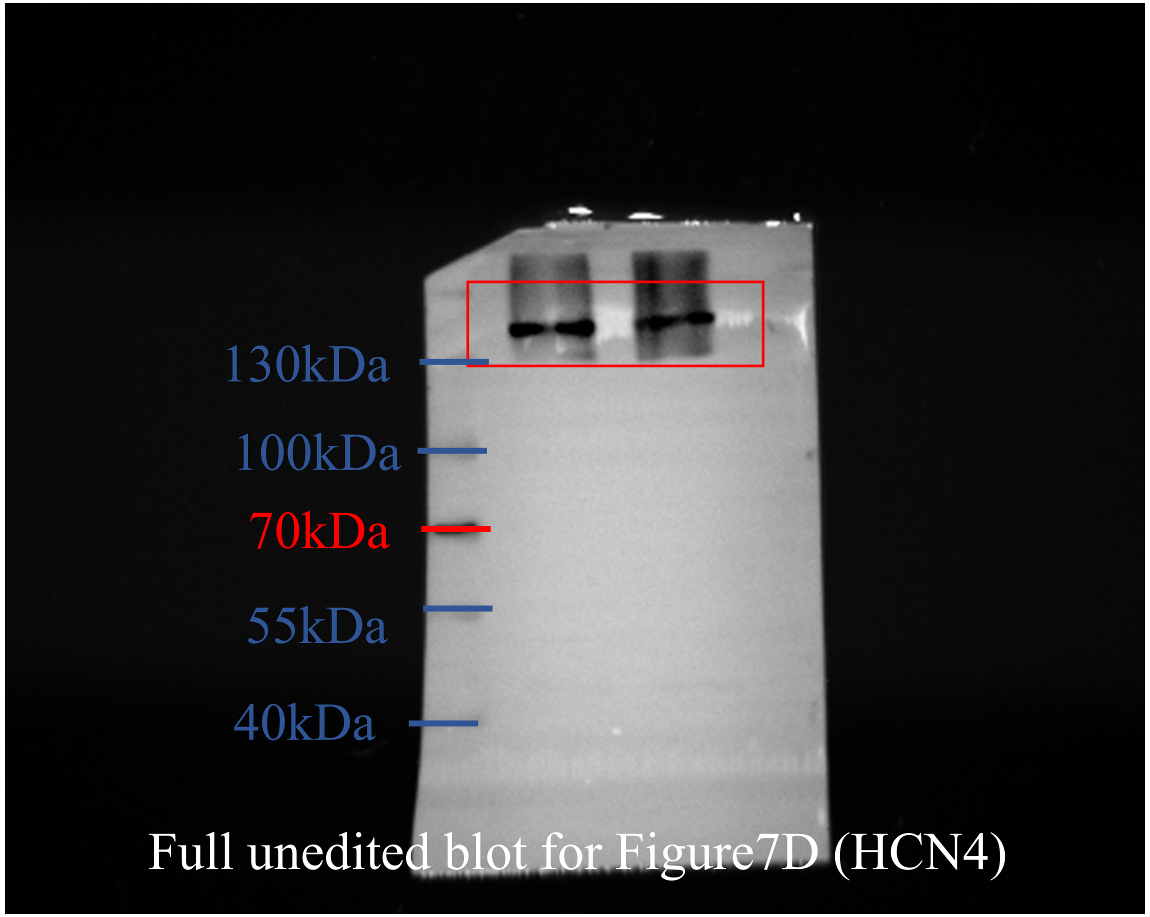

Full unedited blot for Figure7D (HCN4)

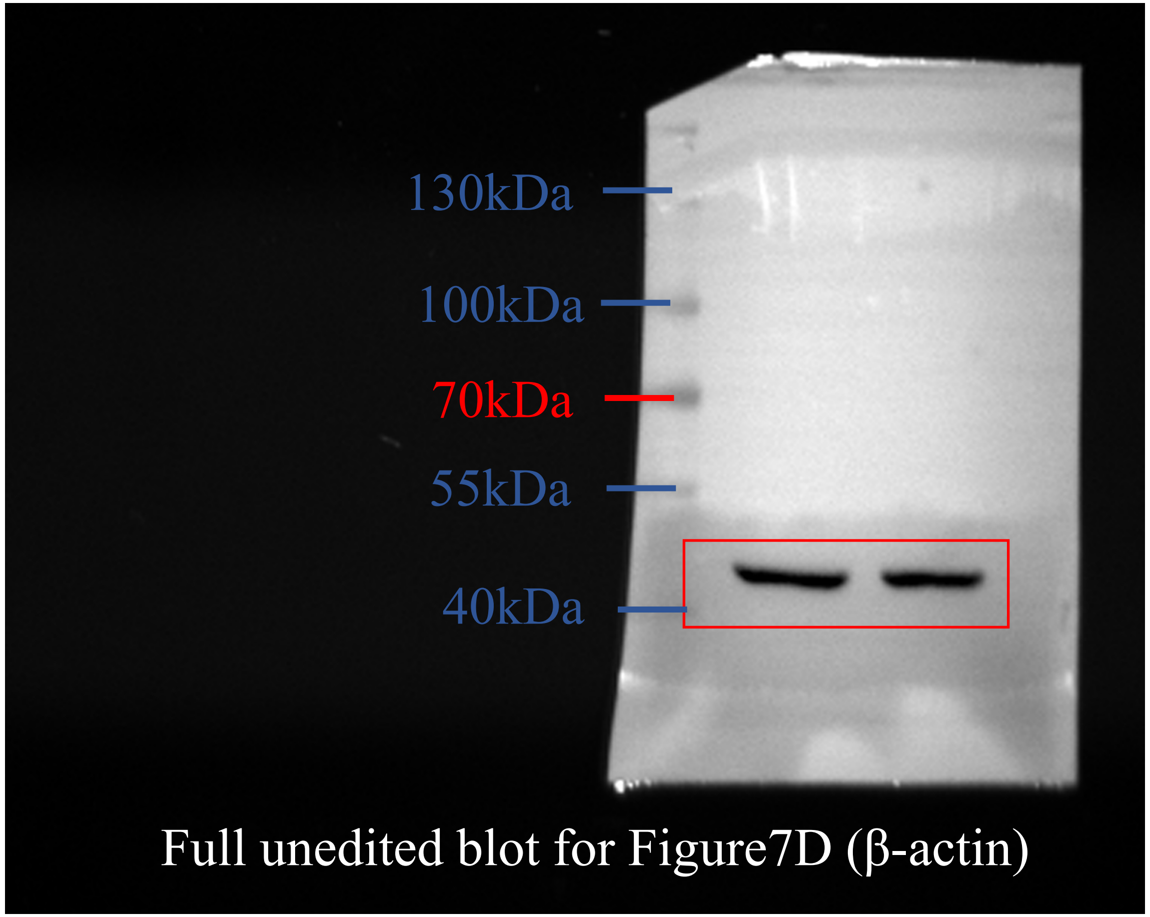

Full unedited blot for Figure7D (β-actin)

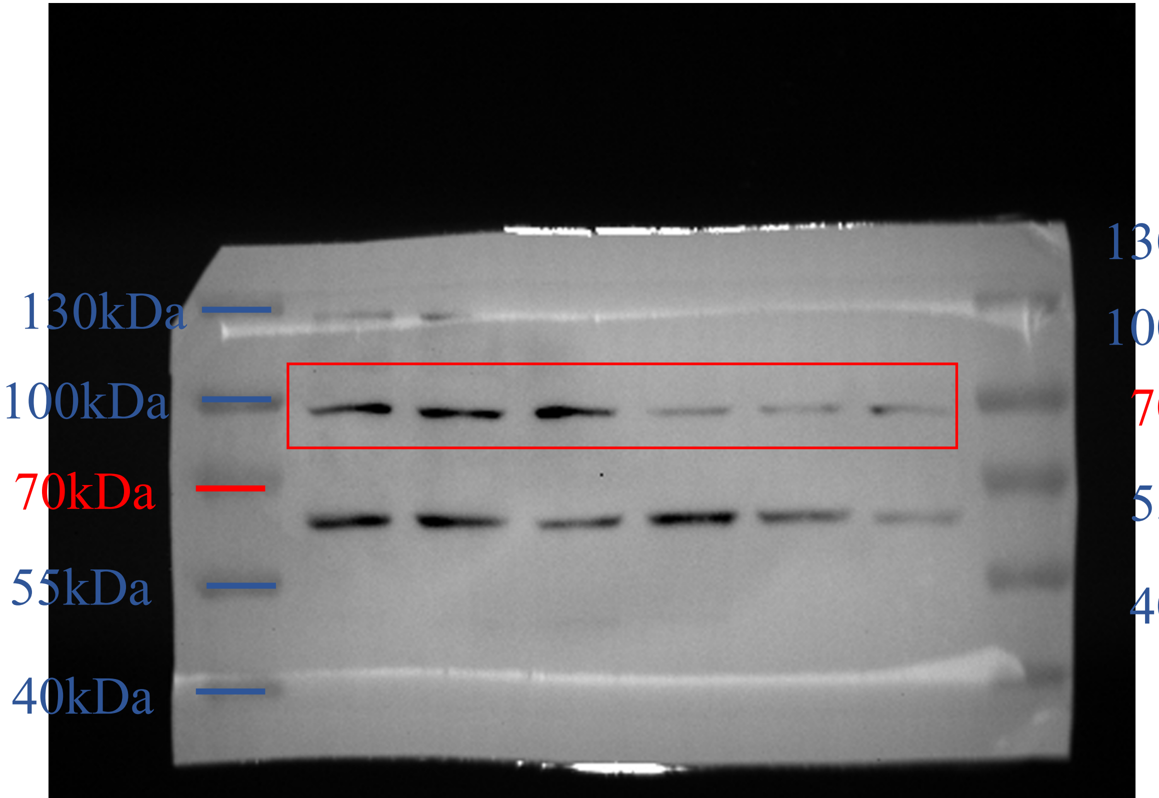

Full unedited blot for Figure10C (HCN2)

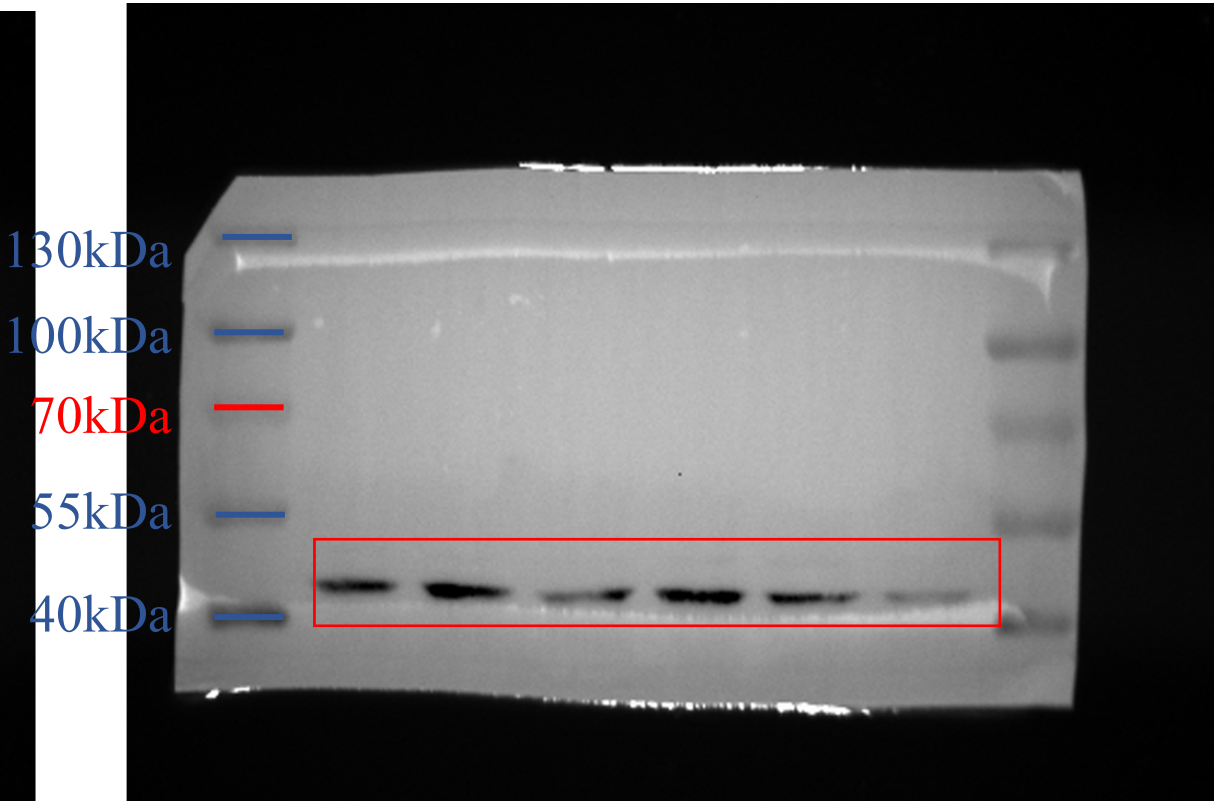

Full unedited blot for Figure10C (β-actin)
