# Supplementary figures and images for: HCN channels in the lateral habenula regulate pain and comorbid depressive‐like behaviors in mice
Source: CNS Neurosci Ther. 2024 Jul 3;30(7):e14831. doi: 10.1111/cns.14831 (PMC11222070; doi:10.1111/cns.14831)

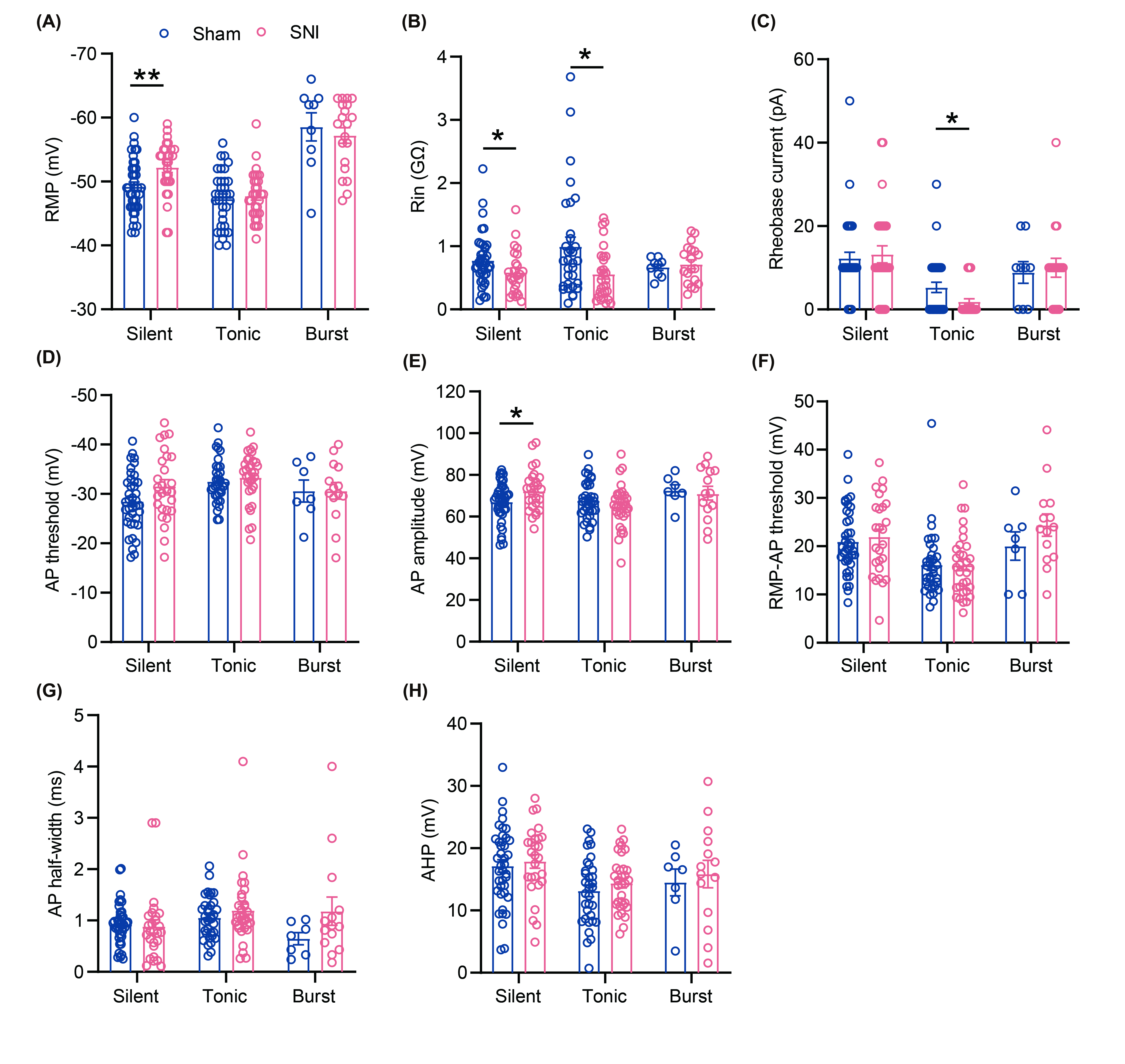

Supplement: Supplementary file 1 — Appendix S1. [file CNS-30-e14831-s001.zip › cns14831-sup-0002-Supplementary_Figure_1.tif]
